# Prenylated Flavonoid Glycosides with PCSK9 mRNA Expression Inhibitory Activity from the Aerial Parts of *Epimedium koreanum*

**DOI:** 10.3390/molecules26123590

**Published:** 2021-06-11

**Authors:** Eeray Kim, Young-Mi Kim, Jongmin Ahn, Hee-Sung Chae, Young-Won Chin, Jinwoong Kim

**Affiliations:** College of Pharmacy and Research Institute of Pharmaceutical Sciences, Seoul National University, Seoul 08826, Korea; kim2546@snu.ac.kr (E.K.); kym0310@snu.ac.kr (Y.-M.K.); jmahn0205@gmail.com (J.A.); chaeheesung83@gmail.com (H.-S.C.); ywchin@snu.ac.kr (Y.-W.C.)

**Keywords:** Herba Epimedii, *Epimedium koreanum*, prenylated flavonoid, PCSK9, LDLR, cholesterol

## Abstract

Phytochemical investigation on the *n*-BuOH-soluble fraction of the aerial parts of *Epimedium koreanum* using the PCSK9 mRNA monitoring assay led to the identification of four previously undescribed acylated flavonoid glycosides and 18 known compounds. The structures of new compounds were elucidated by NMR, MS, and other chemical methods. All isolated compounds were tested for their inhibitory activity against PCSK9 mRNA expression in HepG2 cells. Of the isolates, compounds **6**, **7**, **10**, **15**, and **17**–**22** were found to significantly inhibit PCSK9 mRNA expression. In particular, compound **7** was shown to increase LDLR mRNA expression. Thus, compound **7** may potentially increase LDL uptake and lower cholesterol levels in the blood.

## 1. Introduction

The dried aerial parts of *Epimedium koreanum* Nakai (Berberidaceae), Herba Epimedii, have been used as a tonic or for the treatment of dementia, hypertension, impotence, rheumatic, and paralytic diseases [1,2]. Previous phytochemical studies reported that lignans, phenol glycosides, and prenylated flavonoids are present as chemical constituents of this plant [3,4,5,6]. Individual constituents, including icariin and extracts of *E. koreanum,* demonstrated a variety of biological activities such as anti-hepatotoxic, anti-inflammatory, anti-osteoporosis, anti-tumor, and immunoadjuvant activities, as well as the improvement of sexual function [7,8,9,10,11,12,13].

Proprotein convertase subtilisin/kexin type 9 (PCSK9) is involved in degrading LDLR via clathrin-dependent endocytosis and preventing LDLR recycling, resultantly decreasing the capacity of LDL uptake into cells [14]. Thus, high expression of PCSK9 is often associated with the incidence of hypercholesterolemia, and inhibition of PCSK9 expression or activity has been suggested as a tool to treat patients with familial hypercholesterolemia [15]. Currently, two antibody drugs are prescribed clinically since 2015 [16].

As part of our ongoing project to discover PCSK9 expression inhibitory compounds from medicinal plants [17,18,19,20], the *n*-BuOH-soluble fraction of the aerial parts of *E. koreanum* was selected for further investigation due to its initial PCSK9 mRNA expression inhibitory activity (Supplementary Material Figure S1-1). However, there are no reports regarding PCSK9 inhibitory substances from this plant. Thus, herein, we describe the isolation and identification of four new acylated flavonoid glycosides and 18 known compounds, and their effects on PCSK9 and LDLR mRNA expression in the HepG2 cells.

## 2. Results

### 2.1. Isolation of Compounds from E. koreanum

**Figure 1:**
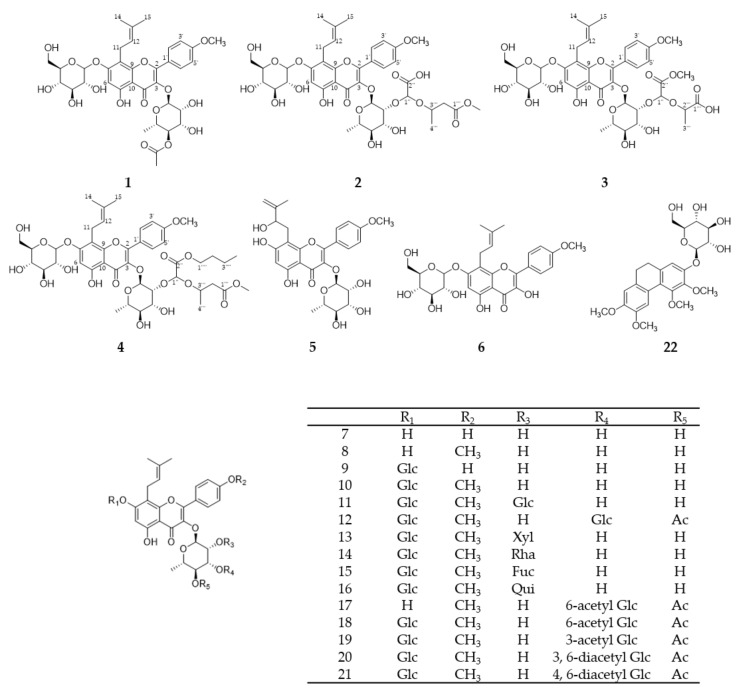
Structures of compounds **1–22**.

The known compounds **5-22** (Figure 1) were confirmed by NMR and MS as koreanoside E (**5**) [21], icariside I (**6**) [22], ikarisoside A (**7**) [23], icariside II (**8**) [24], epimedoside A (**9**) [6,25], icariin (**10**) [26], epimedin A (**11**) [3], korepimedoside C (**12**) [27], epimedin B (**13**) [3], epimedin C (**14**) [3], anhydroicaritin 3-*O*-β-d-fucopyranosyl(1→2)-α-l-rhamnopyranoside-7-*O*-β-d-glucopyranoside (**15**) [28], icarisid I (**16**) [29], korepimedoside A (**17**) [30], epimedokoreanoside I (**18**) [6], korepimeoside C (**19**) [31], epimedin L (**20**) [5], caohuoside B (**21**) [32], and epimedoicarisoside A (**22**) [33].

**Figure 2:**
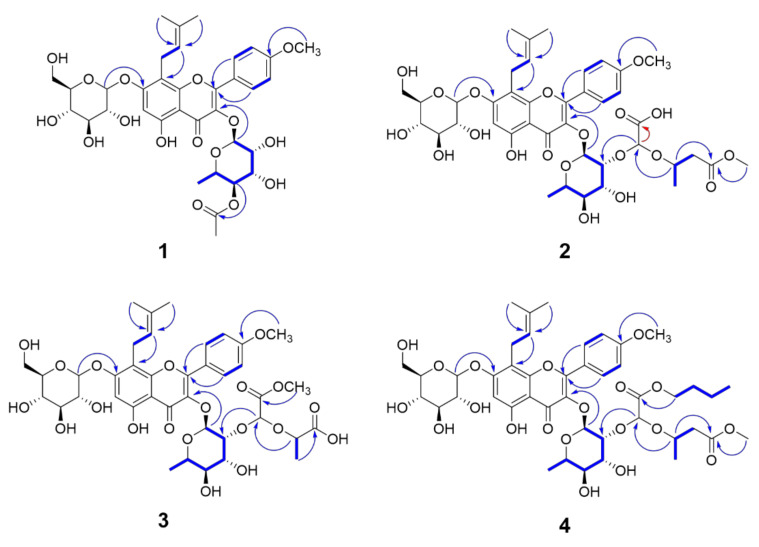
Key ^1^H-^1^H COSY (bold line), HMBC (long range *J* = 8 Hz blue arrow) and HMBC (long range *J* = 2 Hz red arrow) correlations of new compounds.

Compound **1** was obtained as a yellow amorphous powder and its molecular formula was determined to be C_35_H_42_O_16_ by the pseudomolecular ion peak [M + H]^+^ at *m*/*z* 719.2529 (calcd. for C_35_H_43_O_16_, 719.2551) in the positive mode ESI-QTOF-HRMS. In the ^1^H-NMR spectrum of **1** (Table 1), a singlet proton signal at δ_H_ 6.65 (1H, s, H-6), two doublet signals at δ_H_ 7.85 (2H, d, *J* = 8.8 Hz, H-2′ and H-6′) and 7.10 (2H, d, *J* = 8.8 Hz, H- 3′ and H-5′) corresponding to a flavonol skeleton, and the signals responsible for an isoprenyl unit at δ_H_ 3.52 (1H, m, H-11a), 3.57 (1H, m, H-11b), 5.19 (1H, t, *J* = 6.8 Hz, H-12), 1.64 (3H, s, H-14), and 1.73 (3H, s, H-15) were observed. In addition, one methoxy signal at δ_H_ 3.89 (3H, s, 4′-OMe) and the signals for glucose (Glc) with β-conformer (δ_H_ 5.07 (1H, d, *J* = 7.2 Hz, Glc H-1)) and rhamnose (Rha) with α-conformer (δ_H_ 5.50 (1H, brs, Rha H-1)) were detected. All these data indicate that this compound was similar to icariin (**10**), one of the known main constituents in this plant [26]. However, in the ^1^H-NMR and ^13^C-NMR spectroscopic data of **1**, there were additional singlet proton signals at δ_H_ 2.01 (3H, s) and an ester carbon signal at δ_C_ 172.3 belonging to an acetyl group. The position of this acetyl group was assigned to C-4 of rhamnose by the HMBC correlations of δ_H_ 4.82 (Rha H-4) to δ_C_ 172.3, and δ_H_ 2.01 to δ_C_ 172.3 (Figure 2). Further HMBC correlations of δ_H_ 5.07 (Glc H-1) to δ_C_ 161.0 (C-7) and δ_H_ 5.50 (Rha H-1) to δ_C_ 135.9 (C-3) confirmed the locations of glucose and rhamnose at C-7 and C-3, respectively. The absolute configuration of these sugars was determined as d-glucose and l-rhamnose using HPLC analysis of the acid hydrolysate [34]. Thus, the structure of **1** turned out to be icaritin 3-*O*-[4-*O*-acetyl-α-l-rhamnopyranoside]-7-*O*-β-d-glucopyranoside.

Compound **2**, a yellow amorphous powder, had the molecular formula C_40_H_50_O_20_, supported by the pseudomolecular ion peak [M–H]^−^ at *m*/*z* 849.2815 (calcd. for C_40_H_49_O_20_, 849.2817) in the negative mode ESI-QTOF-HRMS. The ^1^H- and ^13^C-NMR spectroscopic data of **2** were similar to those of icariin (**10**), except for the additional signals derived from the presence of a 2-hydroxy-2-((4-methoxy-4-oxobutan-2-yl)oxy)acetic acid unit, a 2,2-dihyroxyacetic acid moiety, and a methyl 3-hydroxybutanoate moiety. The ^1^H NMR signal at δ_H_ 5.21 (1H, s, H-1″) and ^13^C NMR signals at δ_C_ 101.1 (C-1″) and 170.0 (C-2″) were assignable to the 2,2-dihyroxyacetic acid moiety, which was supported by the HMBC correlation (optimized at long range *J* = 2.0 Hz) between H-1″ (δ_H_ 5.21, s) and C-2″ (δ_C_ 170.0). The remaining signals for a methyl 3-hydroxybutanoate moiety were assigned by the sequential correlations of H-2‴ (δ_H_ 2.60 and 2.46)/H-3‴(δ_H_ 4.18, sextet, *J* = 6.0 Hz)/H-4‴ (δ_H_ 1.21, d, *J* = 6.4 Hz) in the ^1^H-^1^H COSY spectrum, and the HMBC correlations of both H-3‴ and a methoxy signal at δ_H_ 3.65 to C-1‴ (δ_C_ 173.1). The connectivity between the 2,2-dihyroxyacetic acid moiety and the methyl 3-hydroxybutanoate moiety was confirmed by the HMBC correlation of H-3‴ (δ_H_ 4.18) to C-1″ (δ_C_ 101.1), constructing 2-hydroxy-2-((4-methoxy-4-oxobutan-2-yl)oxy)acetic acid unit. This 2-hydroxy-2-((4-methoxy-4-oxobutan-2-yl)oxy)acetic acid unit was linked to Rha C-2 via an ether linkage by observing HMBC correlation of H-1″ to δ_C_ 80.0 (Rha C-2). However, the absolute configurations of C-1″ and C-3‴ were not resolved in this study. Therefore, the structure of compound **2** was determined to be icaritin 3-*O*-[2-*O*-2-((4-methoxy-4-oxobutan-2-yl)oxy)acetic acid-α-L-rhamnopyranoside]-7-*O*-β-d-glucopyranoside.

The molecular formula of compound **3** was assigned to be C_39_H_48_O_20_ by pseudomolecular ion peak [M–H]^−^ at *m*/*z* 835.2672 (calcd. for C_39_H_47_O_20_, 835.2661) in the negative mode ESI-QTOF-HRMS. The ^1^H and ^13^C-NMR spectra of **3** were similar to those of **2** except for the presence of a 2-hydroxypropanoic acid moiety instead of the methyl 3-hydroxybutanoate moiety in **2**. The signals responsible for the 2-hydroxypropanoic acid moiety were observed at δ_H_ 4.53 (1H, quintet, *J* = 6.8 Hz H-2‴), and 1.39 (3H, d, *J* = 6.8 Hz H-3‴); δ_C_ 174.4 (C-1‴), 73.2 (C-2‴), and 18.8 (C-3‴). The connectivity of **3** was further confirmed by the HMBC correlations of the methoxy signal at δ_H_ 3.78 to C-2″, H-2‴ to C-1″(δ_C_ 100.4), H-1″(δ_H_ 5.25) to Rha C-2 (δ_C_ 80.0), and a long-range COSY correlation of H-1″ to the methoxy signal. The absolute configuration of sugars was determined as D-glucose and L-rhamnose using HPLC analysis of the acid hydrolysate, while the absolute configurations of C-1″ and C-2‴ were not resolved in this study. Accordingly, compound **3** was characterized as icaritin 3-*O*-[2-*O*-2-((4-oxopropan-2-yl)oxy)acetic acid methyl ester-α-l-rhamnopyranoside]-7-*O*-β-d-glucopyranoside.

Compound **4** was isolated as a yellow amorphous powder. Its molecular formula was determined to be C_44_H_58_O_20_ by the pseudomolecular ion peak [M + HCOO]^−^ at *m*/*z* 951.3529 (calcd. for C_45_H_59_O_22_, 951.3498) in the negative mode ESI-QTOF-HRMS. The ^1^H- and ^13^C-NMR spectra of **4** resembled those of **2** except for the presence of an additional butyl group. The additional butyl group appeared at δ_H_ 4.16 (2H, m, H-1⁗), 1.66 (2H, m, H-2⁗), 1.42 (2H, m, H-3⁗), and 0.95 (3H, t, *J* = 7.2 Hz, H-4⁗). In addition, the sequential correlations from H-1⁗ to H-4⁗ were observed in the ^1^H-^1^H COSY spectrum. The HMBC correlation between H-1⁗ (δ_H_ 4.16, m) and C-2″ (δ_C_ 169.7) suggested the position of a butyl group to be at C-2″ via an ester linkage. Therefore, The structure of **4** was determined to be icaritin 3-*O*-[2-*O*-2-((4-methoxy-4-oxobutan-2-yl)oxy)acetic acid butyl ester-α-l-rhamnopyranoside]-7-*O*-β-d-glucopyranoside.

### 2.2. Bioactivity Evaluation

**Figure 3:**
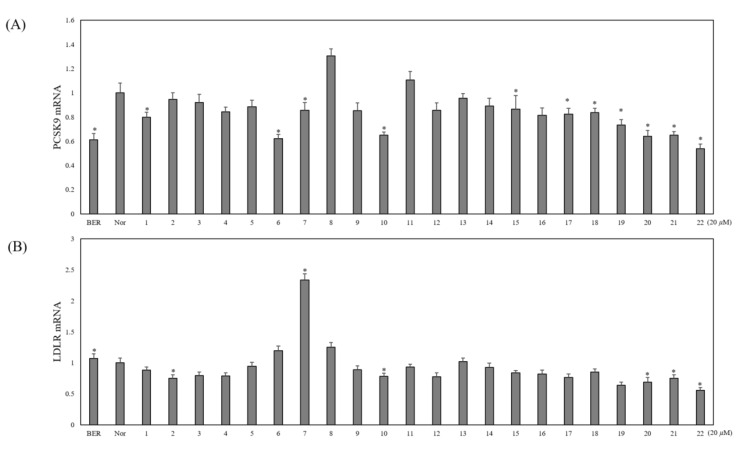
Effects of compounds **1**−**22** and berberine·HCl (BER) on PCSK9 and LDLR regulation in the HepG2 human hepatocellular liver carcinoma cell line. The mRNA expressions of PCSK9 (**A**) and LDLR (**B**) were assayed by qRT-PCR in cells treated with compounds **1**−**22** for 24 h. * *p* < 0.05.

All isolates (**1**–**22**) were tested for their PCSK9 and LDLR mRNA expression in the HepG2 cells. As shown in the Figure 3, compounds **6**, **7**, **10**, **15**, and **17**–**22** were found to inhibit PCSK9 mRNA expression significantly while other flavonoid glycosides seemed to be inactive. Of the active compounds, compound **7** (ikarisoside A) also significantly increased LDLR mRNA expression. Thus, it seems that compound **7** may have potential to increase LDL uptake and lower cholesterol levels in the blood.

## 3. Discussion and Conclusions

The uptake of LDL-cholesterol into the hepatocytes may control cholesterol levels in the blood; this LDL-cholesterol uptake is mediated by LDLR. Hence, adequate LDLR expression in the cells may clear cholesterol in the blood. PCSK9 facilitates the degradation of LDLR after endocytosis of the PCSK-LDLR complex. Upon endocytosis of LDLR-LDL in the absence of PCSK9, LDLR usually dissociates with LDL in the endosomes and then moves back to the cell surface; meanwhile, in the presence of PCSK9, LDLR is degraded in the lysosomes and, resultantly, less LDLR in the cell surface appear, leading to a decrease in the uptake of LDL into cells [35]. Recently, two antibody drugs which interfere the binding of PCSK9 and LDLR were approved for cholesterol-lowering drugs. However, due to some adverse effects of these antibody drugs, small molecules from synthetic molecules or natural molecules were pursued as PCSK9 inhibitory substances [36]. In particular, small molecules from natural sources were found to participate in inhibiting PCSK9 transcriptional or translational expression, PCSK9 secretion, and interaction of PCSK9 and LDLR [37,38]. In this study, PCSK9 transcriptional expressions by the compounds **6**, **7**, **10**, **15**, and **17**–**22** isolated from *E. koreanum* were significantly downregulated. Concomitantly, LDLR transcriptional expression was upregulated by ikarisoside A (**7**). Previously, prenylated flavonoids [20] were able to downregulate PCSK9 expression, but their upregulation of LDLR expression was not documented. As natural compounds with downregulation of PCSK9 expression and upregulation of LDLR expression, α-mangostin [37] and sauchinone [38] were reported and demonstrated an increase in LDL uptake, implying the potential in lowering blood cholesterol. Likewise, ikarisoside A (**7**) may have the positive potential for a cholesterol-lowering effect. Thus, ikarisoside A (**7**) may have strong merits for further investigation in vitro and in vivo.

## 4. Materials and Methods

### 4.1. General Experimental Procedures

Optical rotations were measured using a Jasco P-2000 digital polarimeter (Jasco, Tokyo, Japan). UV spectra were recorded on a UV-VIS spectrometer lamda 25 (Perkin Elmer, Waltham, MA, USA). IR spectra were recorded using Jasco FT/IR-4200 spectrophotometer. Waters Xevo G2 Q-TOF, (Waters, Milford, MA, USA) spectra were measured on a Q-TOF mass spectrometer.

One-dimensional (^1^H and ^13^C) and two-dimensional (^1^H-^1^H COSY, HSQC, HMBC, NOESY) NMR spectra were obtained with a Jeol 400, 600 (JEOL, Tokyo, Japan)—400, 600MHz and Bruker 500 (Bruker, AVANCE 500, Billerica, MA, USA)—500MHz. Column chromatography was performed on silica gel (60-200μm, Zeochem, Switzerland) and diaion HP20 (Mitsubishi chemical, Tokyo, Japan). TLC analysis was run on silica gel 60 F_254_ plates (Merck, Darmstadt, Germany) and visualization of the TLC plates was performed under UV radiation and spraying with 10% aqueous H_2_SO_4_. High-performance liquid chromatography (HPLC) was performed on a Gilson 305/306 pump, equipped with a Gilson UV/VIS 151 detector. Luna 5μ C18 column 250 × 21.20 mm (Phenomenex) and Synergi 4μ hydro-RP column 250 × 21.20 mm (Phenomenex) as HPLC columns were used. Medium-pressure liquid chromatography (MPLC) was run on Isolera One (Biotage, Cardiff, UK). LC grade acetonitrile (MeCN) were purchased from SK Chemicals (Seoul, Korea). Water was purified using a Milli-Q system (Millipore, Bedford, MA, USA). l-cysteine methyl ester hydrochloride and *O*-tolylisothiocyanate were purchased from Tokyo Chemical Industry (Tokyo, Japan).

### 4.2. Plant Material

The aerial parts of *E. koreanum* were purchased from Daerim Pharmaceutical Wholesale Company (Cheongju, Korea) and identified by one of the authors (J. Kim). A voucher specimen (CYWSNUKP-00019) was deposited at the medicinal plant garden in the College of Pharmacy, Seoul National University.

### 4.3. Extraction and Isolation

The air-dried aerial parts of *E. koreanum* (2.0 kg) were extracted with MeOH at room temperature, giving the crude extract (238 g). The crude extract was suspended in water and partitioned successively with *n*-hexane, CHCl_3_, and *n*-BuOH. The *n*-BuOH fraction (94.2 g) was subjected to Diaion HP-20 column chromatography eluted with 20, 40, 60, 80, 100% MeOH to give five fractions (Bu20- Bu100). Bu80 (21.1 g) was subjected to silica gel column chromatography eluted with gradient mixtures of CH_2_Cl_2_/MeOH/H_2_O (50:5:1–7:5:1) and gave 10 subfractions (Bu80.1– Bu80.10). A solid precipitate was separated from Bu80.9 and recrystallized from MeOH to give compound **10** (2.5 g).

Bu80.3 (275 mg) was subjected to reversed-phase (RP) medium pressure liquid chromatography (25 g) and eluted with MeOH/H_2_O (4:6–10:0, step-gradient system, 20 mL/min) to give five fractions (Bu80.3.1- Bu80.3.5). Bu80.3.3 (89.9 mg) was purified using a HPLC column (Luna 5μ C18, 250 × 21.20 mm) and isocratic elution with 41% aqueous MeCN (4 mL/min) to afford compounds **5** (12 mg), **2** (18.7 mg) and subfraction Bu80.3.3.2 (15.7 mg). HPLC purification (Luna 5μ C18, 250 × 21.20 mm, 37% aqueous MeCN, 4 mL/min) of Bu80.3.3.2 furnished compound **3** (3.4 mg).

Bu80.6 (342.1 mg) was subjected to RP-MPLC column chromatography (25 g) using gradient mixtures of MeOH/H_2_O (3:7–10:0, 20 mL/min) to give three fractions (Bu80.6.1- Bu80.6.3). Bu80.6.2 (148.3 mg) was separated by HPLC (Luna 5μ C18, 250 × 21.20 mm) and eluted with 41% aqueous MeCN (4 mL/min), furnishing compounds **21** (5.5 mg), **7** (8.4 mg) and subfraction Bu80.6.2.2 (49.3 mg). From Bu80.6.2.2, compound **20** (29.7 mg) was purified by HPLC (Luna 5μ C18, 250 × 21.20 m, 4 mL/min m) using isocratic elution of 37% aqueous MeCN.

Bu80.8 (475.1 mg) was fractionated into four subfractions (Bu80.8.1- Bu80.8.4) by RP-MPLC (25 g), and eluted with gradient mixtures of MeOH/H_2_O (3:7–10:0, 20 mL/min). Bu80.8.2 (322.8 mg) was subjected to HPLC separation (Luna 5μ C18, 250 × 21.20 mm) and eluted with 39% aqueous MeCN (4 mL/min) to give subfractions Bu80.8.2.2 (33.2 mg) and Bu80.8.2.3 (27.5 mg). Compound **18** (28.3 mg) was isolated from Bu80.8.2.2 by HPLC separation (Synergi 4μ hydro-RP, 250 × 21.20 mm, 35% aqueous MeCN, 4 mL/min). From Bu80.8.2.3, compound **19** (17.4 mg) was purified by HPLC (Synergi 4μ hydro-RP, 250 × 21.20 mm, 4 mL/min) and isocratically eluted with 35% aqueous MeCN.

Bu80.10 (8.5 g) was subjected to RP-MPLC (50 g) using gradient mixtures of MeOH/H_2_O (3:7–10:0, 40 mL/min) to give 8 fractions (Bu80.10.1- Bu80.10.8). Bu80.10.3 (405.5 mg) was purified using HPLC (Synergi 4μ hydro-RP, 250 × 21.20 mm, 25% aqueous MeCN, 8 mL/min) to give compound **9** (14.7 mg). Bu80.10.4 (5.8 g) was separated by HPLC (Synergi 4μ hydro-RP, 250 × 21.20 mm) and eluted with 29% aqueous MeCN (8 mL/min) to obtain compounds **11** (19.4 mg), **13** (32.8 mg), **14** (29.5 mg), **15** (7.1 mg), **16** (3.6 mg) and **12** (7.6 mg).

Bu100 (16.9 g) was subjected to silica gel column chromatography and eluted with gradient mixtures of CH_2_Cl_2_/MeOH/H_2_O (50:5:1–7:5:1) to give 10 fractions (Bu100.1–Bu100.10). Bu100.4 (1.1 g) was separated into four fractions (Bu100.4.1- Bu100.4.4) by RP-MPLC and eluted with gradient mixtures of MeOH/H_2_O (4:6-10:0, 40 mL/min). From Bu100.4.1 (46.3 mg), compound **22** (4.4 mg) was isolated by HPLC separation (Synergi 4μ hydro-RP, 250 × 21.20 mm, 28% aqueous MeCN, 8 mL/min). HPLC purification (Synergi 4μ hydro-RP, 250 × 21.20 mm, 42% aqueous MeCN, 8 mL/min) of Bu100.4.3 (402.3 mg) furnished compounds **6** (16.5 mg), **8** (66.7 mg), **4** (8.2 mg) and **17** (21.3 mg).

Bu100.7 (1.4 g) was subjected to RP-MPLC (50 g) using gradient mixtures of MeOH/H_2_O(4:6–10:0, 40 mL/min) to give four fractions (Bu100.7.1–Bu100.7.4). From Bu100.7.2 (552.9 mg), compound **1** (12.5 mg) was purified using HPLC (Synergi 4μ hydro-RP, 250 × 21.20 mm) using isocratic elution of 22% aqueous MeCN (8 mL/min).

### 4.4. Characterization

(**1**): Yellow amorphous powder; ${\left[\text{α}\right]}_{\text{D}}^{25}$ -137.2 (*c* 0.1, MeOH); UV (MeOH) λ_max_ nm (log ε) 228 (3.28), 264 (3.23), 314 (2.79), 353 (2.40); IR (KBr) ν_max_ 3409, 2923, 1647, 1597, 1261 cm^−1^; ^1^H-NMR (400 MHz) and ^13^C-NMR (100 MHz) data, see Table 1; HRMS (ESI-TOF) *m*/*z* 719.2529 (calcd. for C_35_H_43_O_16_, 719.2551).

(**2**): Yellow amorphous powder; ${\left[\text{α}\right]}_{\text{D}}^{20}$ -79.3 (*c* 0.1, MeOH); UV (MeOH) λ_max_ nm (log ε) 267 (1.25), 313 (0.70), 344 (0.56); IR (KBr) ν_max_ 3383, 2931, 1738, 1654, 1596, 1511, 1437, 1376, 1342, 1303, 1259, 1220, 1181, 1143 cm^−1^; ^1^H-NMR (400 MHz) and ^13^C-NMR (100 MHz) data, see Table 1; HRMS (ESI-TOF) *m*/*z* 849.2815 (calcd. for C_40_H_49_O_20_, 849.2817).

(**3**): Yellow amorphous powder; ${\left[\text{α}\right]}_{\text{D}}^{20}$ -56.1 (*c* 0.1, MeOH); UV (MeOH) λ_max_ nm (log ε) 267 (1.01), 313 (0.57), 342 (0.47); IR (KBr) ν_max_ 2924, 1748, 1595, 1508, 1489, 1339, 1259, 1181 cm^−1^; ^1^H-NMR (400 MHz) and ^13^C-NMR (100 MHz) data, see Table 1; HRMS (ESI-TOF) *m*/*z* 835.2672 (calcd. for C_39_H_47_O_20_, 835.2661).

(**4**): Yellow amorphous powder; ${\left[\text{α}\right]}_{\text{D}}^{20}$ -65.2 (*c* 0.1, MeOH UV (MeOH) λ_max_ nm (log ε) 267 (1.15), 313 (0.64), 345 (0.53); IR (KBr) ν_max_ 3414, 2932, 1739, 1653, 1597, 1511, 1438, 1375, 1304, 1259, 1219, 1181 cm^−1^; ^1^H-NMR (400 MHz) and ^13^C-NMR (100 MHz) data, see Table 1; HRMS (ESI-TOF) *m*/*z* 951.3529 (calcd. for C_45_H_59_O_22_, 951.3498).

### 4.5. Acid Hydrolysis

Compounds were hydrolyzed using 1 N H_2_SO_4_ (200 µL) and heated with a water bath at 90 °C for 2 h, then neutralized with saturated aqueous Na_2_CO_3_ solution. After the solutions were dried under a stream of N_2_, the products and standard sugars (d-Glc, l-Rha) were dissolved in pyridine (200 µL) containing D-cysteine methyl ester hydrochloride (1 mg). After that, they were heated at 60 °C for 1 h. The solutions were treated with 2 µL (1.11 mg) of *O*-tolylisothiocyanate and then heated again at 60 °C for 1 h. Each final mixture was directly analyzed by analytical RP-HPLC (Hypersil™ BDS C18 column, 150 × 4.60 mm, 25% aqueous. MeCN, 0.8 mL/min). The peaks at 19.20 and 31.92 min of the derivatives of d-glucose and l-rhamonse, respectively, were coincided with the peaks of the derivatives of d-glucose and l-rhamnose in compounds **1**–**4**.

### 4.6. Cell Culture, Drugs and Chemicals

HepG2 (human hepatocellular liver cell line) was obtained from the Korea Research Institute of Bioscience and Biotechnology (Daejeon, Korea) and grown in Eagle’s minimum essential medium (EMEM), supplemented with 10% fetal bovine serum and 100 U/mL penicillin/streptomycin sulfate. Cells were incubated in a humidified incubator at 37 °C in a 5% CO_2_ atmosphere. EMEM, penicillin, and streptomycin were purchased from HyClone Laboratories (Logan, UT, USA). Oligonucleotide primers for LDLR, PCSK9, and GAPDH were purchased from Bioneer Corp. (Daejeon, Korea). Berberine·HCl was purchased from Chengdu Biopurify Phytochemicals Ltd. (Sichuan, China).

### 4.7. Quantitative Real-Time RT-PCR

Total cellular RNA was isolated using a Trizol RNA extraction kit (Thermo Fisher Scientific, Waltham, MA, USA) according to the manufacturer’s instructions. Total RNA (1 μg) was then converted to cDNA using 200 units of iScript cDNA Synthesis Kit (Bio-Rad, Hercules, CA, USA) at 25 °C for 5 min and at 46 °C for 20 min. The reaction was stopped by incubating the solution at 95 °C for 1 min, after which 1 μL of cDNA mixture was used for enzymatic amplification. PCR reactions were performed using 4 μL of the cDNA and 6 μL master mix containing iQ SYBR Green Supermix (Bio-Rad), 5 pmol of forward primer, and 5 pmol of reverse primer, in a CFX96 real-time PCR detection system (Bio-Rad). Reaction conditions were 3 min at 95 °C, followed by 40 cycles of 10 s at 95 °C and 30 s at 55 °C. The plate was read subsequently. The fluorescence signal generated with SYBR Green I DNA dye was measured during the annealing step. The specificity of the amplification was confirmed using a melting curve analysis. Data were collected and recorded with CFX Manager Software (Bio-Rad) and expressed as a function of the threshold cycle (CT). The relative quantity of the gene of interest was then normalized to the relative quantity of GAPDH (ΔΔCT). The mRNA abundance in the sample was calculated using the 2−(ΔΔCT) method. The following specific primer sets were used (5′ to 3′): human GAPDH: GAAGGTGAAGGTCGGAGTCA (forward), AATGAAGGGGTCATTGATGG (reverse); human LDLR: GTGCTCCTCGTCTTCCTTTG (forward), TAGCTGTAGCCGTCCTGGTT (reverse); human PCSK9: GGTACTGACCCCCAACCTG (forward), CCGAGTGTGCTGACCATACA (reverse). Gene-specific primers were custom-synthesized by Bioneer (Daejeon, Korea).

### 4.8. Statistical Analysis

For multiple comparisons, one-way analysis of variance (ANOVA) was performed followed by Dunnett’s *t* test. Data from experiments are presented as means ± standard error of the mean. The number of independent experiments analyzed is given in the figure captions. P-values of less than 0.05 were regarded as statistically significant.

## Figures and Tables

**Table 1:** ^1^H and ^13^C-NMR Data of compounds **1**-**4** (CD_3_OD).

Position	1		2		3		4	
	δ_H_ (*J* in Hz)	δ_C_	δ_H_ (*J* in Hz)	δ_C_	δ_H_ (*J* in Hz)	δ_C_	δ_H_ (*J* in Hz)	δ_C_
2		159.4		159.2		159.4		159.3
3		135.9		136.8		136.6		136.9
4		180.0		180.0		180.0		180.1
5		161.0		161.0		160.9		161.1
6	6.65, s	99.4	6.65, s	99.4	6.67, s	99.9	6.67, s	99.4
7		162.1		162.1		162.1		162.2
8		110.5		110.6		110.7		110.6
9		155.0		155.0		155.0		155.1
10		107.5		107.5		107.5		107.5
11	3.52, m, 3.57, m	22.7	3.51, m, 3.57, m	22.7	3.52, m, 3.58, m	22.7	3.53, m, 3.57, m	22.8
12	5.19, t (6.8)	123.5	5.18, m	123.6	5.20, m	123.4	5.19, m	123.6
13		132.6		132.7		132.8		132.7
14	1.64, s	25.9	1.64, s	25.9	1.64, s	25.9	1.64, s	25.9
15	1.73, s	18.3	1.72, s	18.3	1.75, s	18.3	1.72, s	18.3
1′		123.9		123.8		123.7		123.9
2′, 6′	7.85, d (8.8)	131.9	7.86, d (8.8)	131.9	7.90, d (8.4)	131.9	7.89, d (8.8)	132.0
3′, 5′	7.10, d (8.8)	115.2	7.08, d (8.8)	115.2	7.10, d (8.4)	115.2	7.10, d (8.8)	115.3
4′		163.6		163.5		163.6		163.6
4’-OMe	3.89, s	56.1	3.89, s	56.1	3.90, s	56.1	3.89, s	56.1
Glucose								
1	5.07, d (7.2)	101.9	5.07, d (6.8)	101.9	5.07, d (7.2)	101.8	5.07, d (7.2)	101.9
2	3.53, m	74.9	3.53, m	74.9	3.54, m	74.9	3.53, m	74.9
3	3.51, m	78.2	3.51, m	78.3	3.52, m	78.3	3.51, m	78.4
4	3.43, m	71.1	3.43, m	71.1	3.43, m	71.1	3.42, m	71.2
5	3.48, m	78.3	3.48, m	78.2	3.48, m	78.2	3.49, m	78.3
6	3.92, m 3.74, m	62.4	3.92, m 3.74, m	62.4	3.92, m 3.74, m	62.3	3.92, m 3.74, m	63.4
Rhamnose								
1	5.50, brs	102.7	5.45, d (1.6)	102.2	5.45, d (2.0)	102.0	5.45, brs	102.2
2	4.21, brs	71.7	4.33, brs	80.0	4.36, brs	80.0	4.34, brs	79.9
3	3.85, dd (9.8, 3.0)	70.0	3.80, dd (9.2, 3.2)	71.9	3.92, m	72.2	3.80, dd (9.6, 3.2)	71.9
4	4.82, t (10.0)	74.9	3.38, m	73.3	3.41, m	71.7	3.39, m	73.4
5	3.23, m	69.6	3.33, m	72.2	3.34, m	72.9	3.29, m	72.2
6	0.77, d (6.0)	17.5	0.95, d (6.0)	17.7	0.94, d (6.4)	17.6	0.95, d (5.2)	17.7
4-O-Ac		172.3						
	2.01, s	20.9						
Terminal								
1″			5.21, s	101.1	5.25, s	100.4	5.21, s	101.3
2″				170.0		169.4		169.7
2″-OMe					3.78, s	52.9		
1‴				173.1		174.4		173.1
2‴			2.46, m 2.60, m	42.5	4.53, q (6.8)	73.2	2.46, m 2.62, m	42.6
3‴			4.18, sextet (6.0)	73.3	1.39, d (6.8)	18.8	4.18, m	73.4
4‴			1.21, d (6.4)	21.6			1.21, d (6.0)	21.7
1‴-OMe			3.65, s	52.2			3.66, s	52.2
1⁗							4.16, m	66.4
2⁗							1.66, m	31.7
3⁗							1.42, m	20.2
4⁗							0.95, t (7.2)	14.1

^1^H and ^13^C-NMR spectra were obtained from 400 and 100 MHz, respectively.

## Data Availability

Not applicable.

## Supplementary Materials

The following are available online, Figures S1-1–S5-3: NMR, MS, and sugar analysis for new compounds (**1**–**4**) and NMR and MS data for compound 7, Table S5-1: NMR chemical shifts of compound **7**.

## Sample Availability

Samples of the compounds are available from the authors.
