# Supplementary material for: Prenylated Flavonoid Glycosides with PCSK9 mRNA Expression Inhibitory Activity from the Aerial Parts of Epimedium koreanum
Source: Molecules. 2021 Jun 11;26(12):3590. doi: 10.3390/molecules26123590 (PMC8230864; doi:10.3390/molecules26123590)
Supplement: Supplementary file 1 [file molecules-26-03590-s001.zip › molecules-1225952-supplementary.pdf]

SUPPORTING INFORMATION

Article

Prenylated Flavonoid Glycosides with PCSK9 mRNA Expression Inhibitory Activity from the Aerial Parts of *Epimedium koreanum*

Eeray Kim, Young-Mi Kim, Jongmin Ahn, Hee-Sung Chae, Young-Won Chin and Jinwoong Kim *

College of Pharmacy and Research Institute of Pharmaceutical Sciences, Seoul National University, Seoul 08826, Korea
* Correspondence: jwkim@snu.ac.kr; Tel.: +82-2-880-7853

List of Supplementary material:

No.	Content	Page
**Figure S 1-1.**	Effects of extracts of E. koreanum on PCSK9 mRNA expression in the HepG2 human hepatocellular liver carcinoma cell line.	3
**Figure S 2-1.**	The ESIHRMS of **1**	4
**Figure S 2-2.**	The ^{1}H NMR (400 MHz) spectrum of **1** in CD_3OD	5
**Figure S 2-3.**	The ^{13}C NMR (100 MHz) spectrum of **1** in CD_3OD	6
**Figure S 2-4.**	The DEPT-135 (100 MHz) spectrum of **1** in CD_3OD	7
**Figure S 2-5.**	The HSQC spectrum of **1** in CD_3OD	8
**Figure S 2-6.**	The ^{1}H-^{1}H COSY spectrum of **1** in CD_3OD	9
**Figure S 2-7.**	The HMBC spectrum of **1** in CD_3OD	10
**Figure S 2-8.**	The ^{1}H-^{1}H NOESY spectrum of **1** in CD_3OD	11
**Figure S 2-9**	The UV spectrum of **1**	12
**Figure S 2-10.**	Acid hydrolysis of **1**	13
**Figure S 3-1.**	The ESIHRMS of **2**	14
**Figure S 3-2.**	The ^{1}H NMR (400 MHz) spectrum of **2** in CD_3OD	15
**Figure S 3-3.**	The ^{13}C NMR (100 MHz) spectrum of **2** in CD_3OD	16
**Figure S 3-4.**	The DEPT-135 (100 MHz) spectrum of **2** in CD_3OD	17
**Figure S 3-5.**	The HSQC spectrum of **2** in CD_3OD	18
**Figure S 3-6.**	The ^{1}H-^{1}H COSY spectrum of **2** in CD_3OD	19
**Figure S 3-7.**	The HMBC spectrum of **2** in CD_3OD	20
**Figure S 3-8.**	The HMBC (2 Hz) spectrum and expanded HMBC spectrum of **2** in CD_3OD	21-22
**Figure S 3-9**	The ^{1}H-^{1}H NOESY spectrum of **2** in CD_3OD	23
**Figure S 3-10.**	The UV spectrum of **2**	24
**Figure S 3-11.**	Acid hydrolysis of **2**	25
**Figure S 4-1.**	The ESIHRMS of **3**	26
**Figure S 4-2.**	The ^{1}H NMR (400 MHz) spectrum of **3** in CD_3OD	27
**Figure S 4-3.**	The ^{13}C NMR (100 MHz) spectrum of **3** in CD_3OD	28
**Figure S 4-4.**	The DEPT-135 (100 MHz) spectrum of **3** in CD_3OD	29
**Figure S 4-5.**	The HSQC spectrum of **3** in CD_3OD	30
**Figure S 4-6.**	The ^{1}H-^{1}H COSY spectrum of **3** in CD_3OD	31
**Figure S 4-7.**	The HMBC spectrum of **3** in CD_3OD	32
**Figure S 4-8.**	The long-range COSY spectrum of **3** in CD_3OD.	33
**Figure S 4-9.**	The UV spectrum of **3**	34
**Figure S 4-10**	Acid hydrolysis of **3**	35
**Figure S 5-1.**	The ESIHRMS of **4**	36
**Figure S 5-2.**	The ^{1}H NMR (400 MHz) spectrum of **4** in CD_3OD	37
**Figure S 5-3.**	The ^{13}C NMR (100 MHz) spectrum of **4** in CD_3OD	38
**Figure S 5-4.**	The HSQC spectrum of **4** in CD_3OD	39
**Figure S 5-5.**	The ^{1}H-^{1}H COSY spectrum of **4** in CD_3OD	40
**Figure S 5-6.**	The HMBC spectrum of **4** in CD_3OD	41
**Figure S 5-7.**	The ^{1}H-^{1}H NOESY spectrum of **4** in CD_3OD	42
**Figure S 5-8.**	The UV spectrum of **4**	43
**Figure S 5-9**	Acid hydrolysis of **4**	44
**Table S 6-1.**	Assignment of ^{1}H and ^{13}C NMR spectra of **7** in CD_3OD	45
**Figure S 6-1.**	The ESIHRMS of **7**	46
**Figure S 6-2.**	The ^{1}H NMR (400 MHz) spectrum of **7** in CD_3OD	47
**Figure S 6-3.**	The ^{13}C NMR (100 MHz) spectrum of **7** in CD_3OD	48

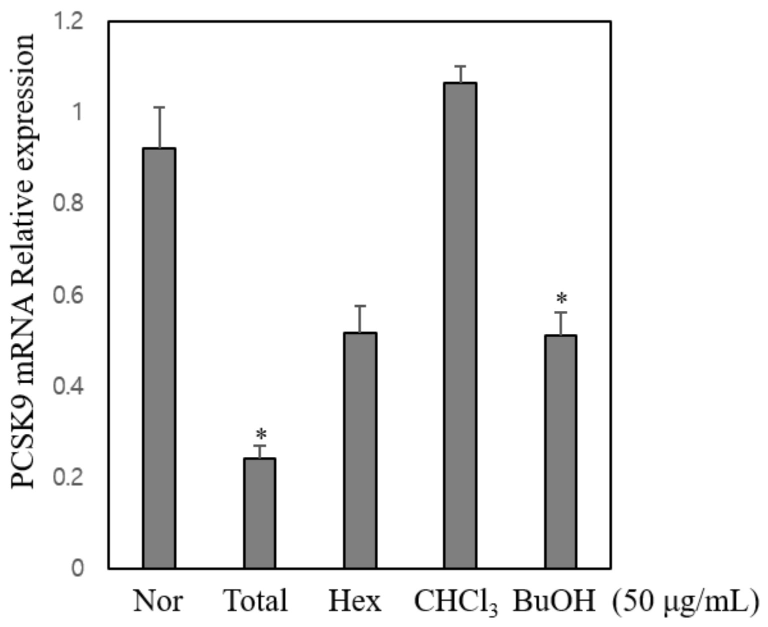

Figure S 1-1. Effects of extracts of *E. koreanum* on PCSK9 mRNA expression in the HepG2 human hepatocellular liver carcinoma cell line.

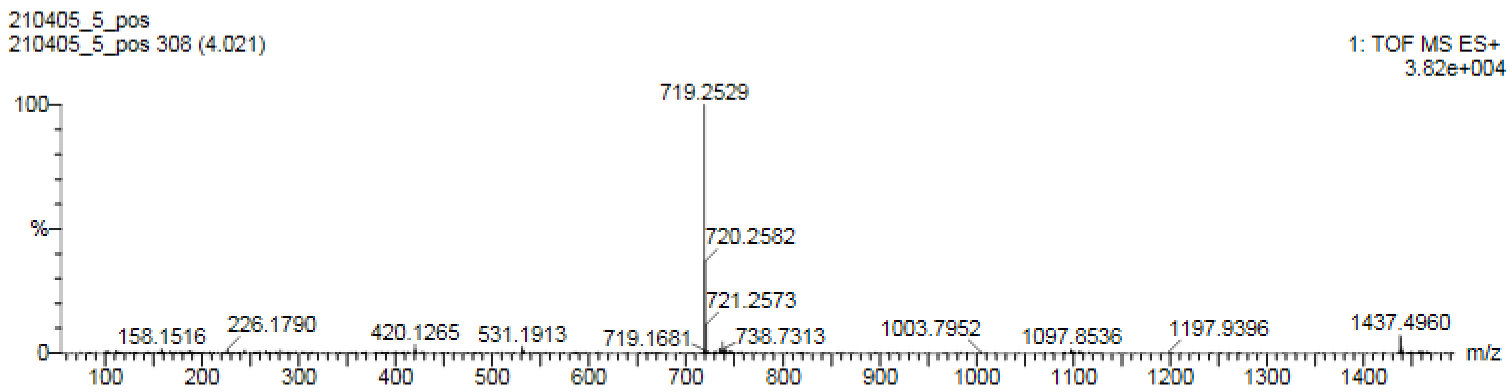

Minimum:				-1.5				
Maximum:		5.0	10.0	50.0				

Mass	Calc. Mass	mDa	PPM	DBE	i-FIT	Norm	Conf(%)	Formula
719.2529	719.2527	0.2	0.3	11.5	125.0	0.845	42.96	C33 H44 O16 Na
	719.2551	-2.2	-3.1	14.5	124.8	0.588	55.53	C35 H43 O16
	719.2562	-3.3	-4.6	33.5	132.7	8.560	0.02	C51 H36 O3 Na
	719.2492	3.7	5.1	23.5	129.9	5.745	0.32	C42 H39 O11
	719.2586	-5.7	-7.9	36.5	133.4	9.251	0.01	C53 H35 O3
	719.2586	-5.7	-7.9	2.5	129.6	5.413	0.45	C26 H48 O21 Na
	719.2468	6.1	8.5	20.5	129.3	5.141	0.59	C40 H40 O11 Na
	719.2457	7.2	10.0	1.5	130.8	6.638	0.13	C24 H47 O24

Figure S 2-1. The ESIHRMS of **1**.

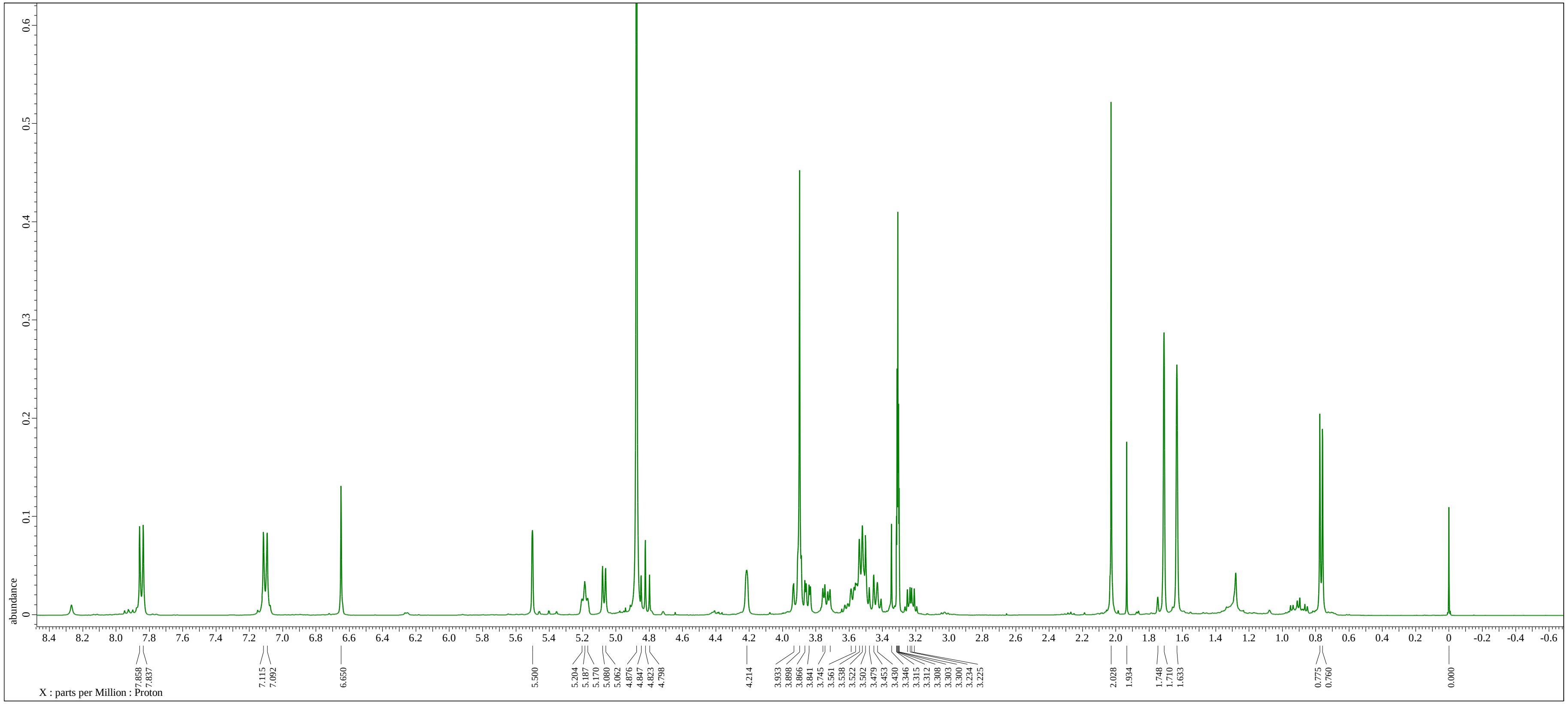

Figure S 2-2. The ^1H NMR (400 MHz) spectrum of **1** in CD_3OD.

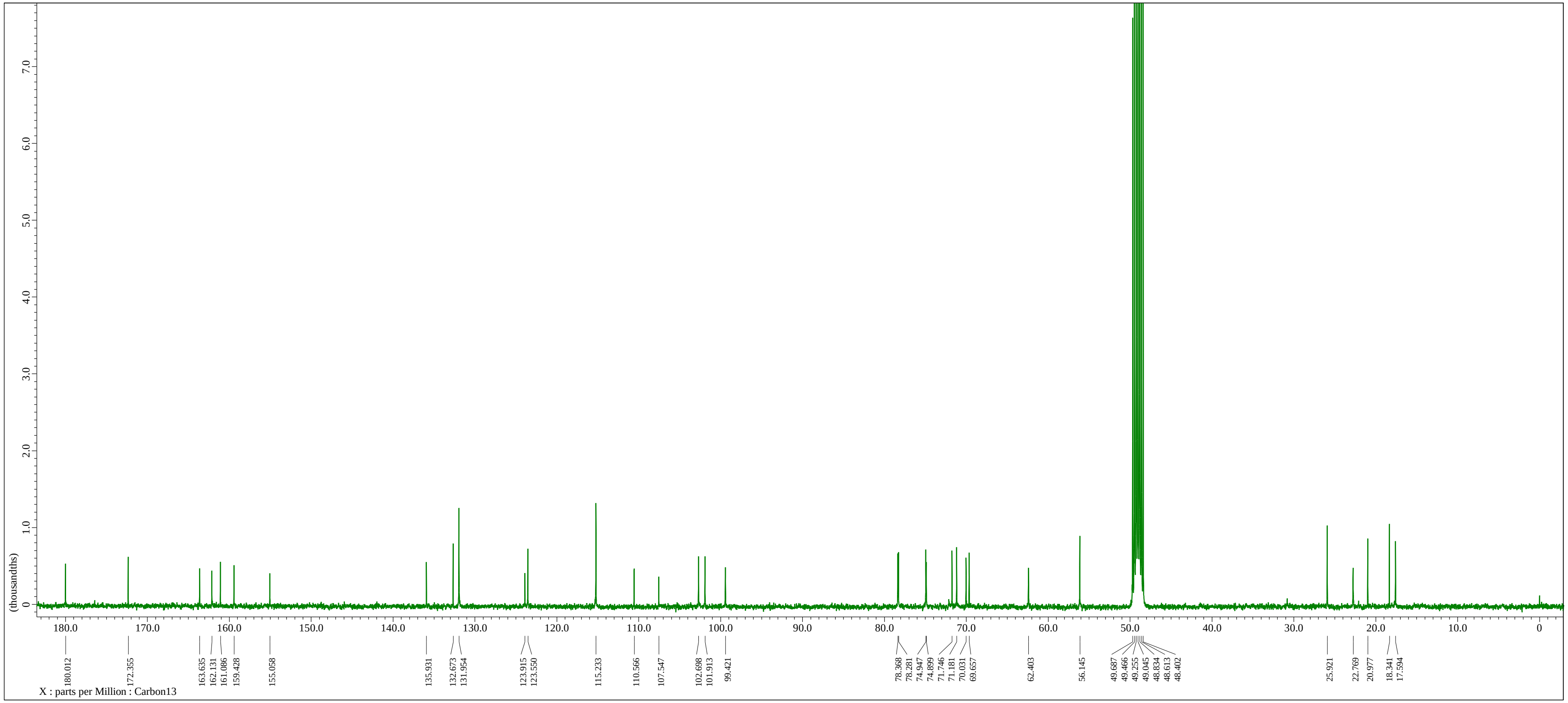

Figure S 2-3. The ^{13}C NMR (100 MHz) spectrum of **1** in CD_3OD.

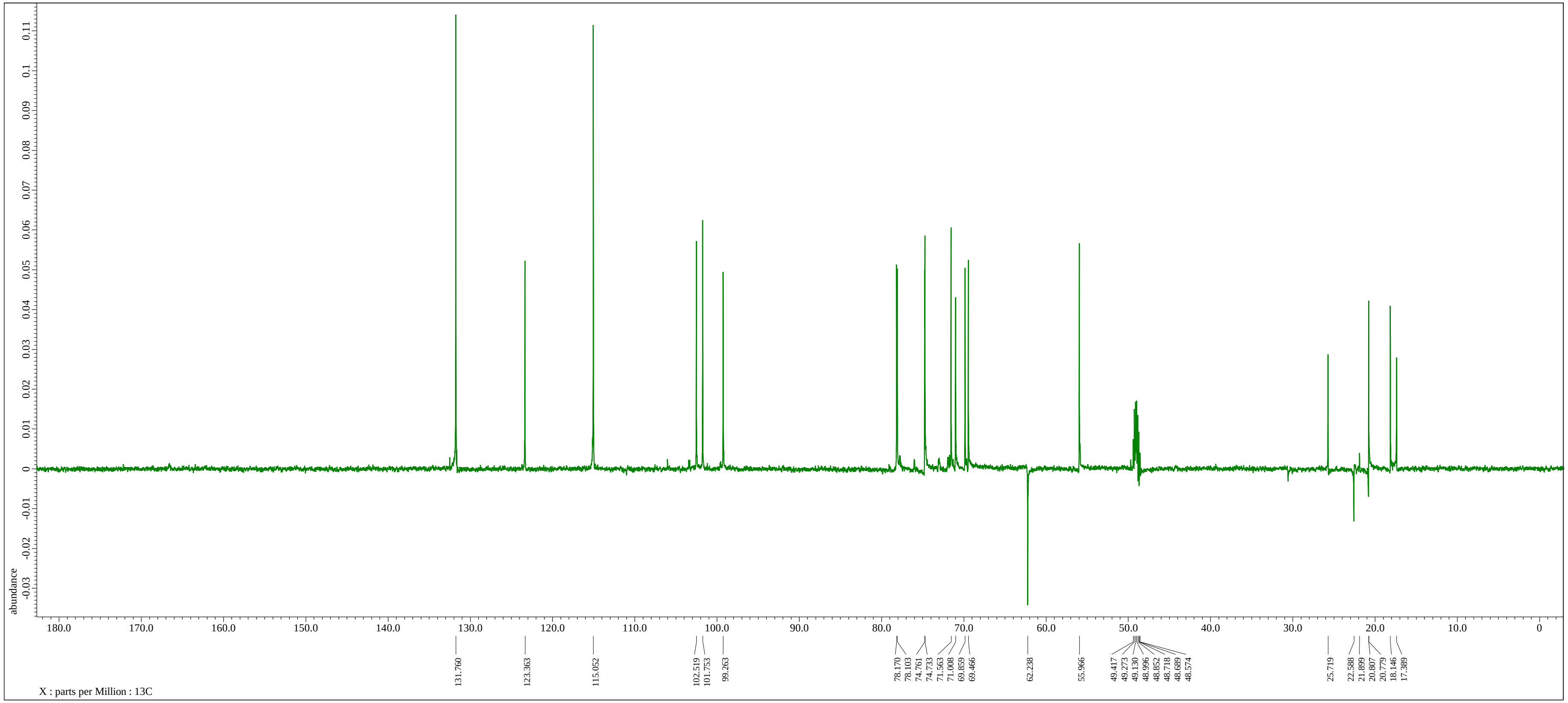

Figure S 2-4. The DEPT-135 (100 MHz) spectrum of **1** in CD_3OD.

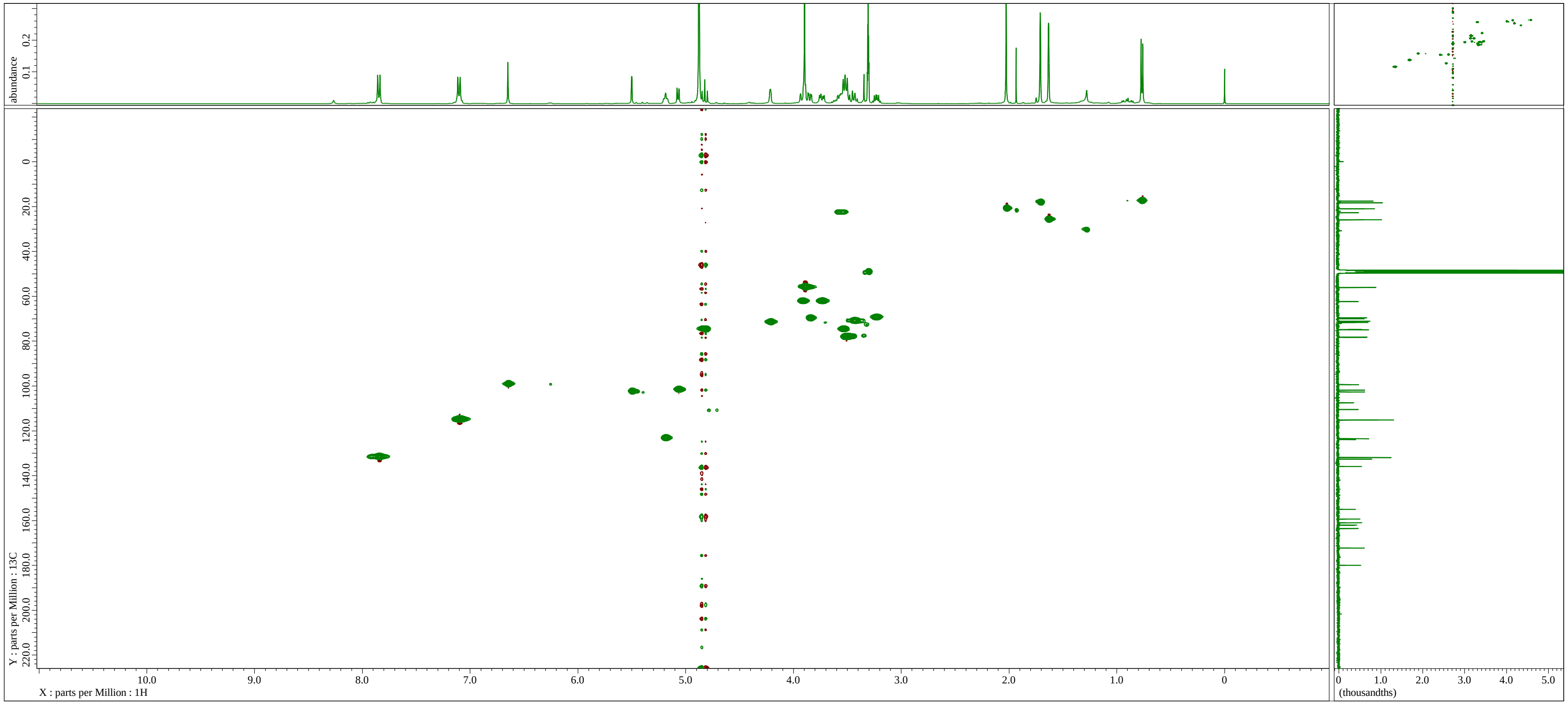

Figure S 2-5. The HSQC spectrum of **1** in CD_3OD

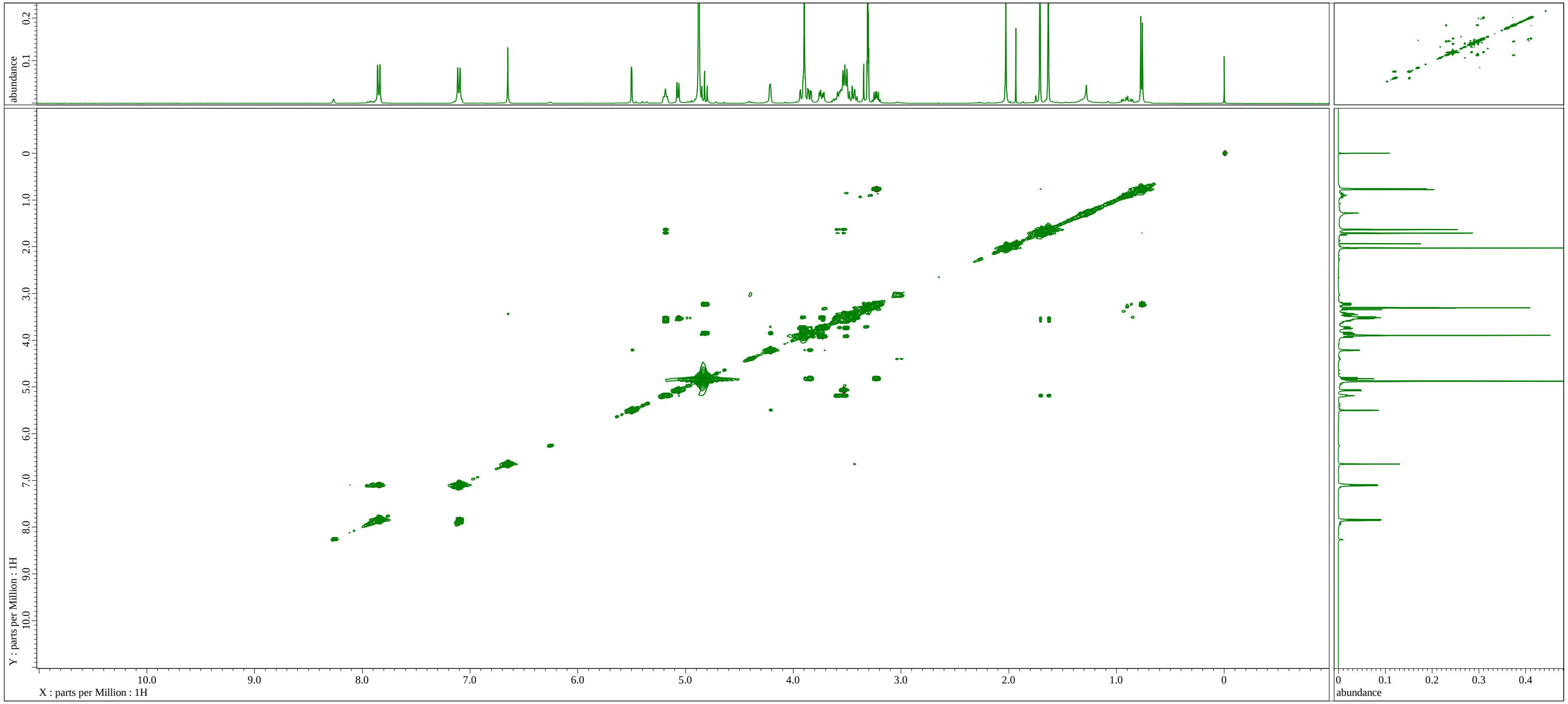

Figure S 2-6. The COSY spectrum of **1** in CD_3OD

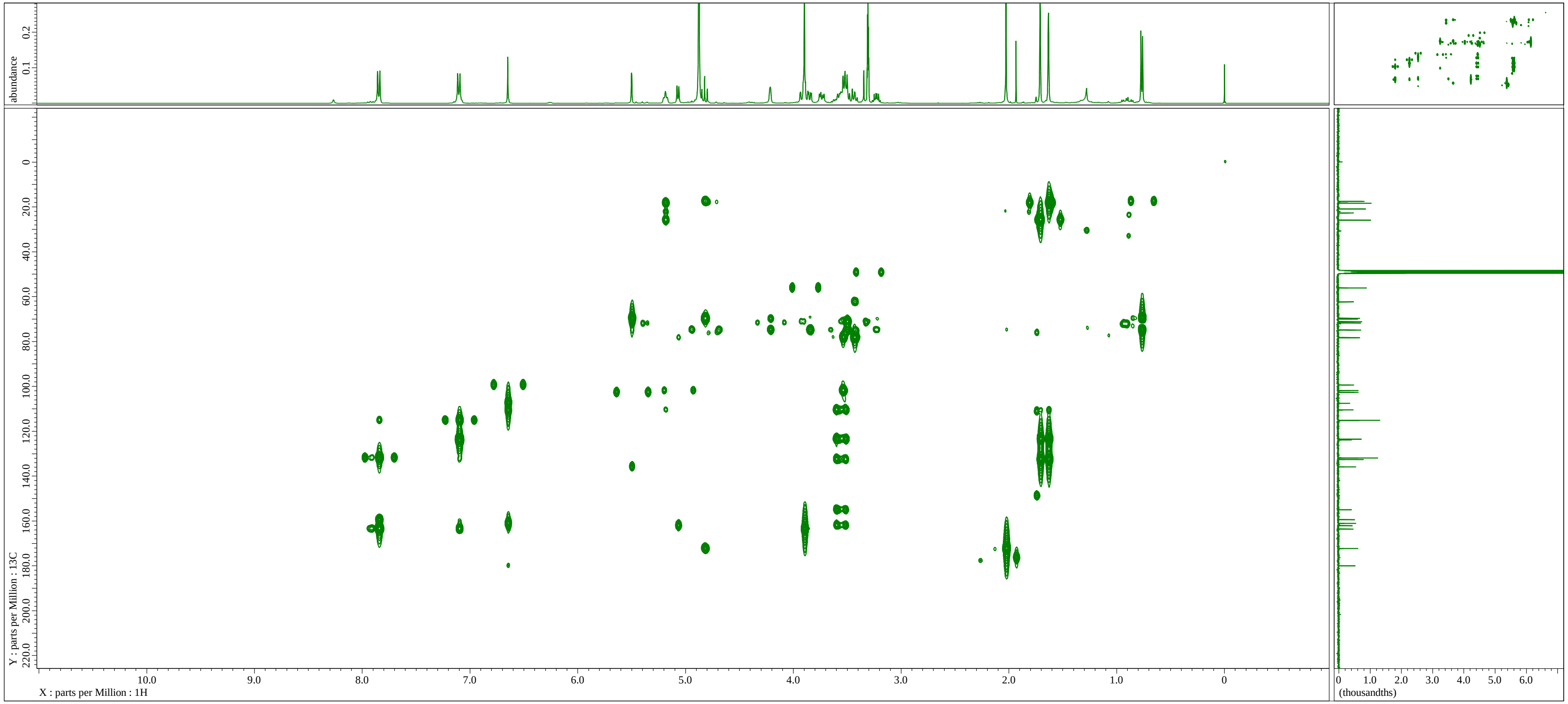

Figure S 2-7. The HMBC spectrum of **1** in CD_3OD

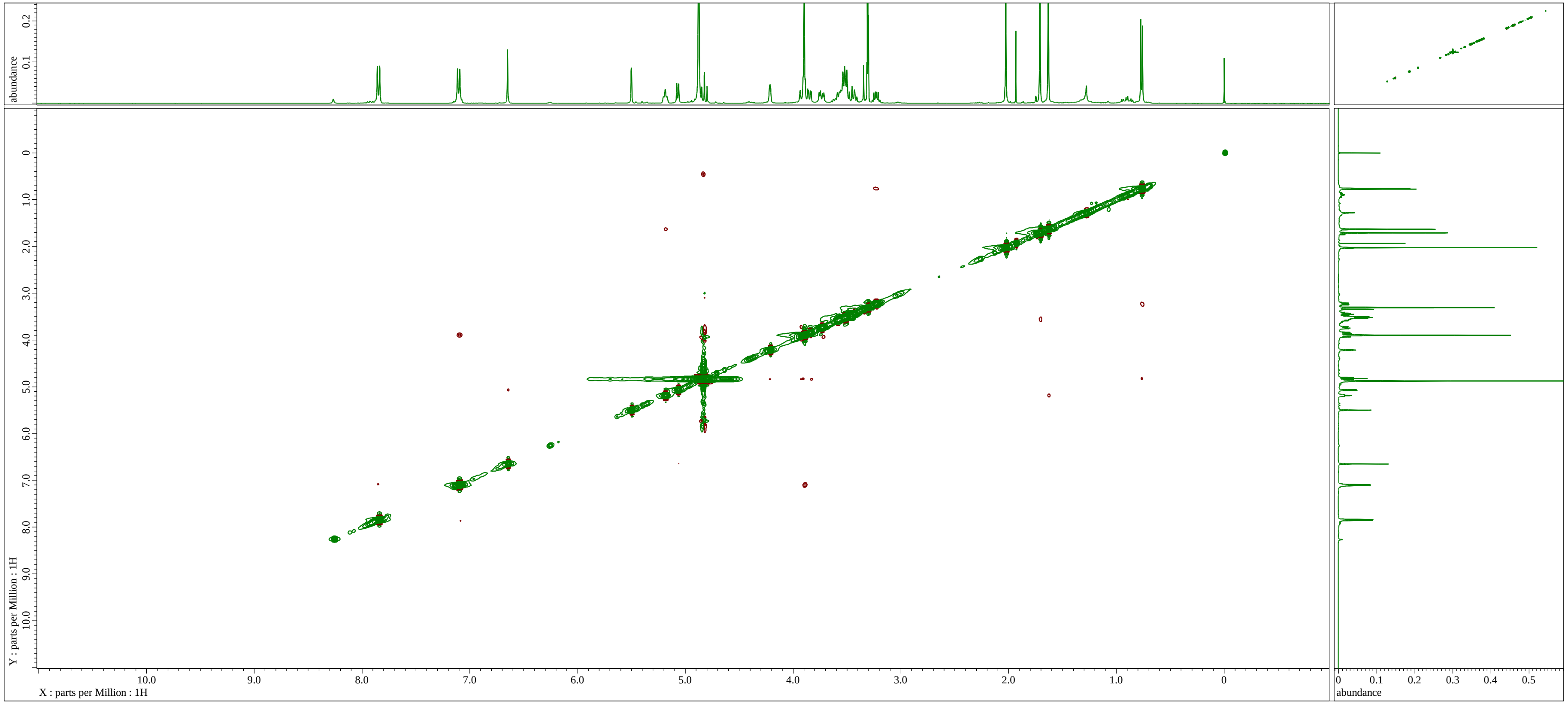

Figure S 2-8. The NOESY spectrum of **1** in CD_3OD

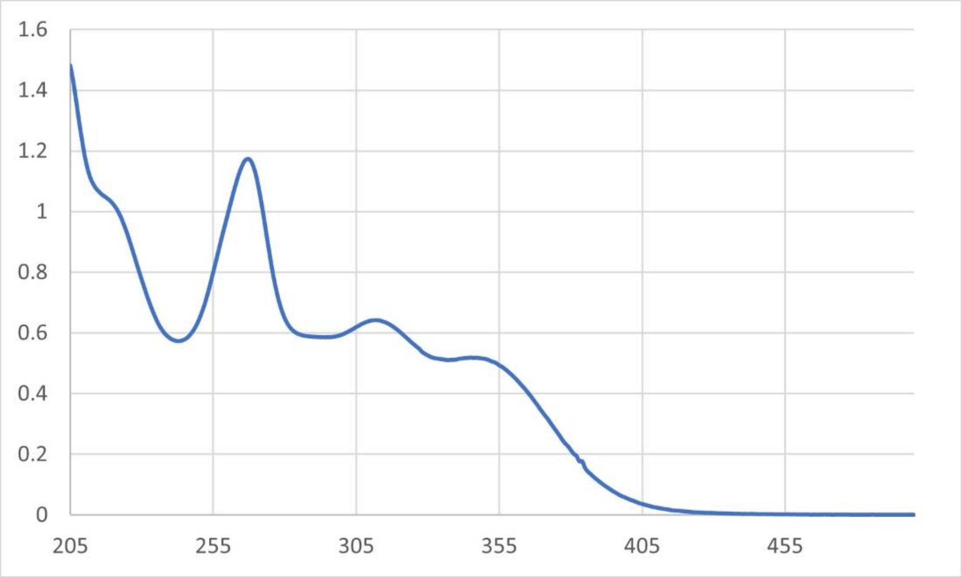

Figure S 2-9. The UV spectrum of **1** in CD_3OD

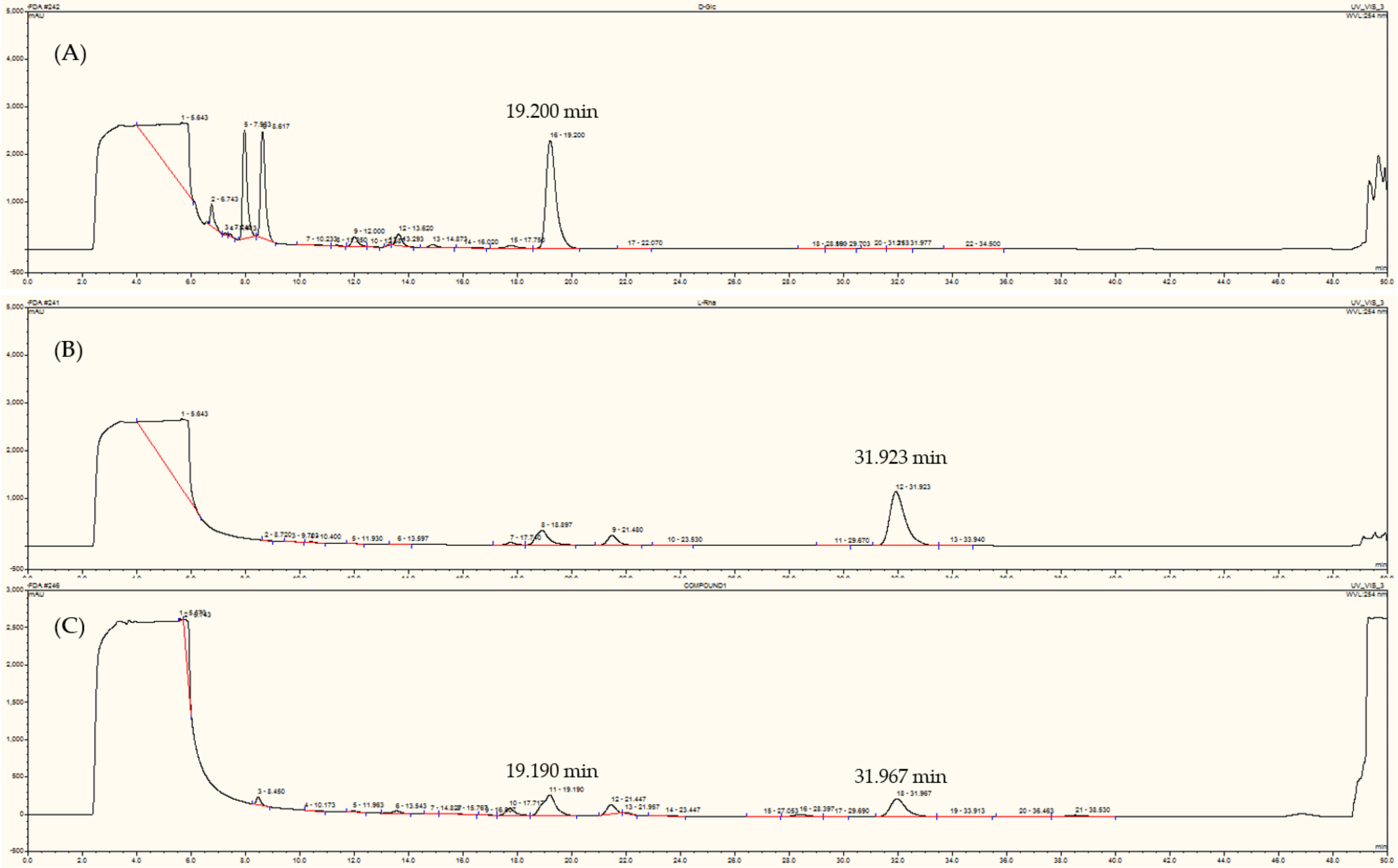

(A) D-glucose derivative (B) L-rhamnose derivative (C) derivatives of acid hydrolysate

Figure S 2-10. Acid hydrolysis of **1.**

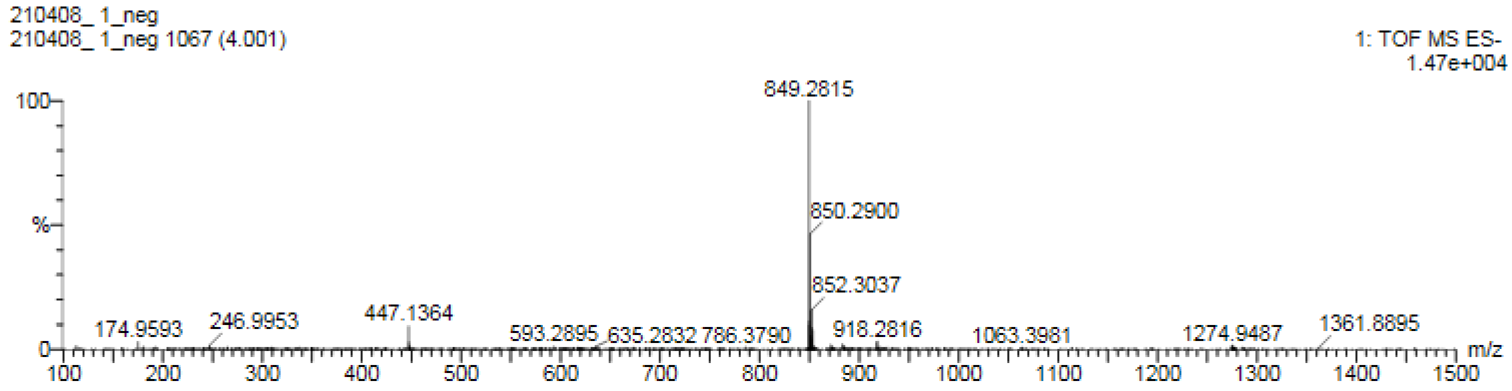

Minimum:				-1.5				
Maximum:		5.0	10.0	50.0				

Mass	Calc. Mass	mDa	PPM	DBE	i-FIT	Norm	Conf(%)	Formula
849.2815	849.2817	-0.2	-0.2	16.5	184.2	0.126	88.14	C40 H49 O20
	849.2758	5.7	6.7	25.5	186.3	2.228	10.78	C47 H45 O15
	849.2876	-6.1	-7.2	7.5	189.0	4.844	0.79	C33 H53 O25
	849.2852	-3.7	-4.4	38.5	190.1	6.026	0.24	C58 H41 O7
	849.2794	2.1	2.5	47.5	191.6	7.486	0.06	C65 H37 O2

Figure S 3-1. The ESIHRMS of **2**.

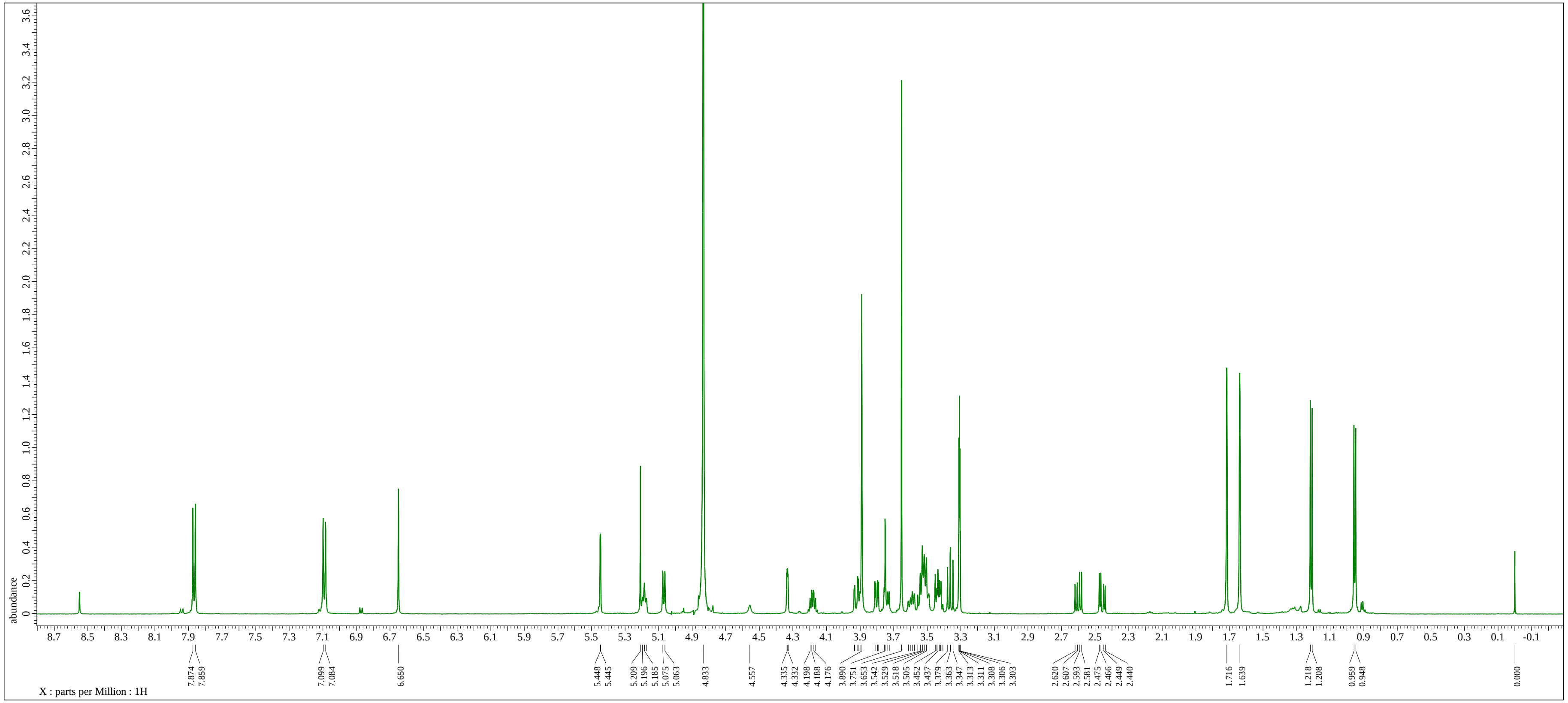

Figure S 3-2. The ^{1}H NMR spectrum of **2** in CD_3OD.

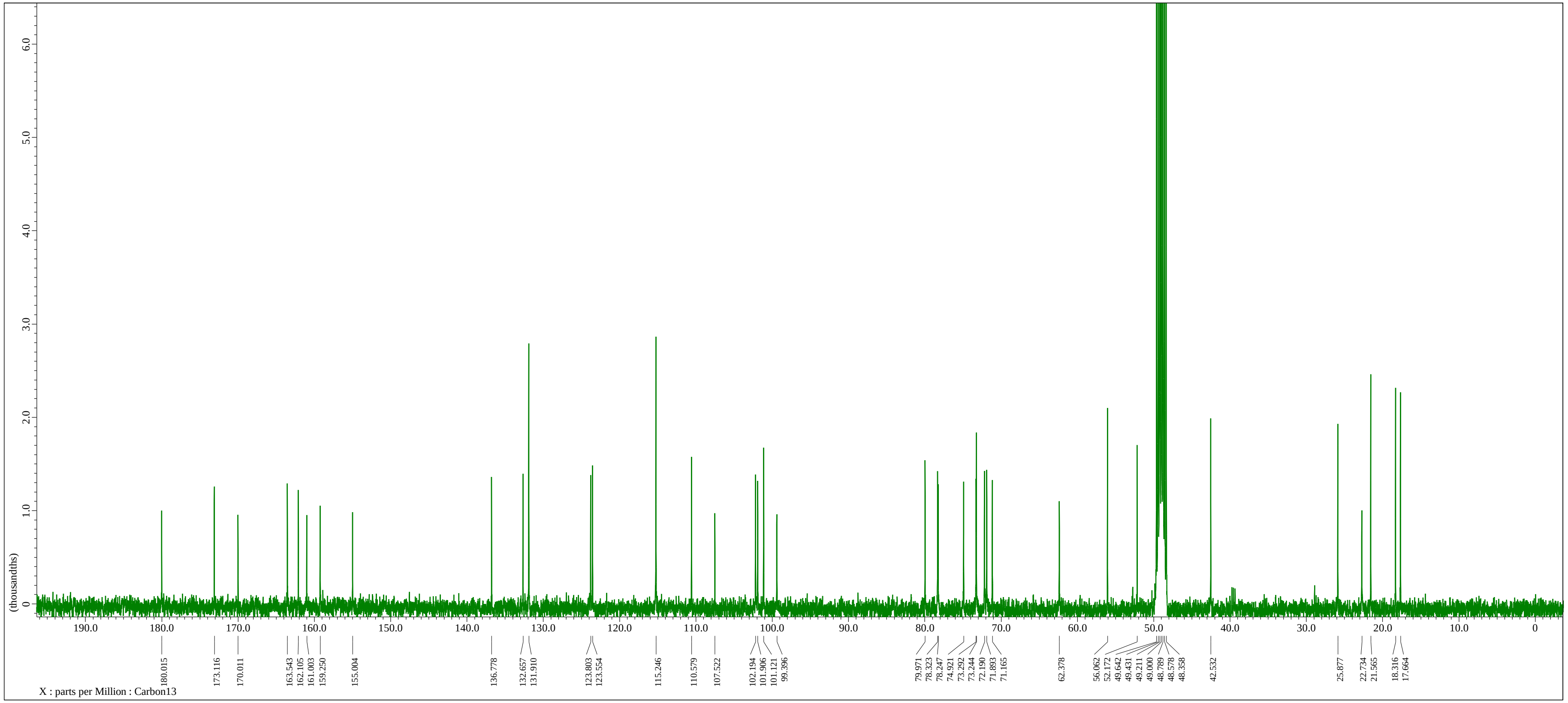

Figure S 3-3. The ^{13}C NMR spectrum of **2** in CD_3OD.

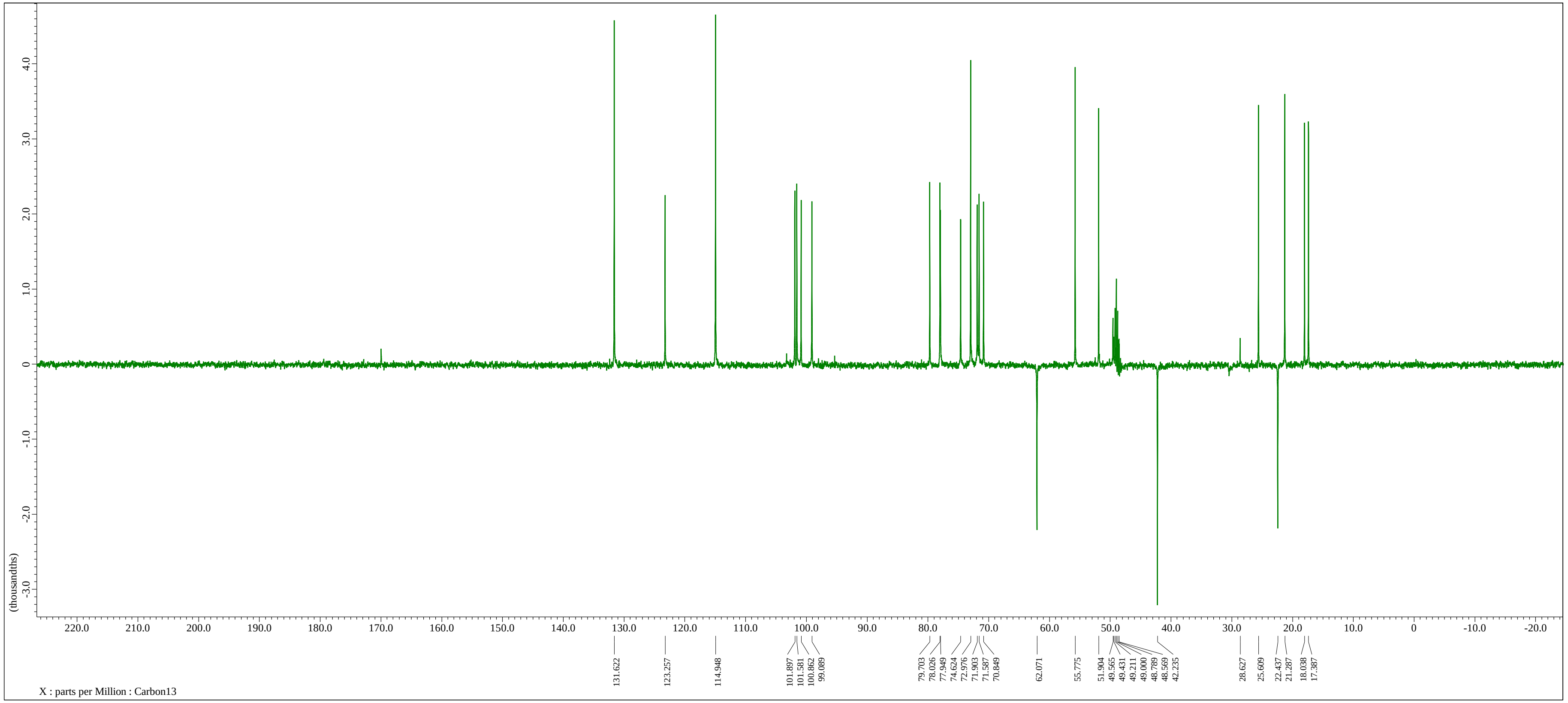

Figure S 3-4. The DEPT-135 (100 MHz) spectrum of **2** in CD_3OD.

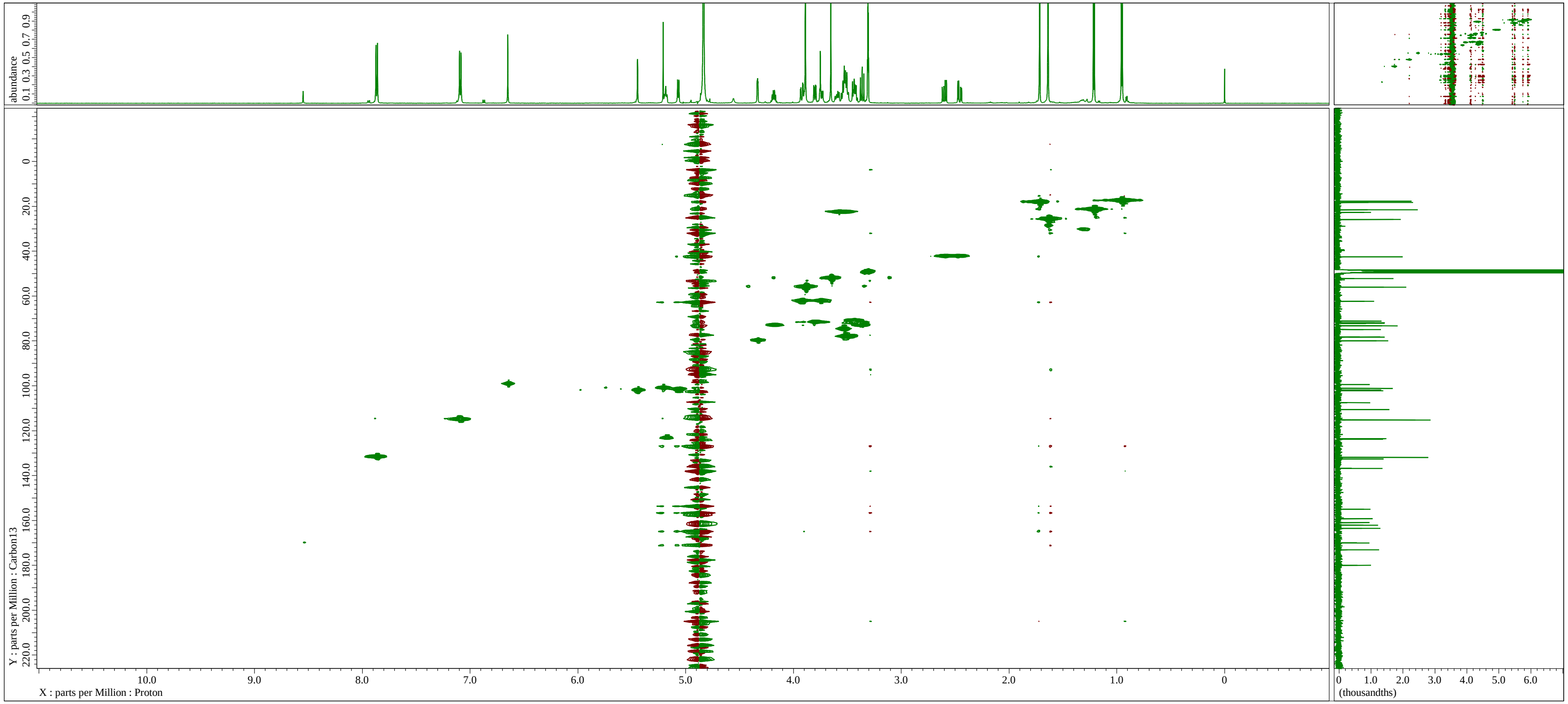

Figure S 3-5. The HSQC spectrum of **2** in CD_3OD.

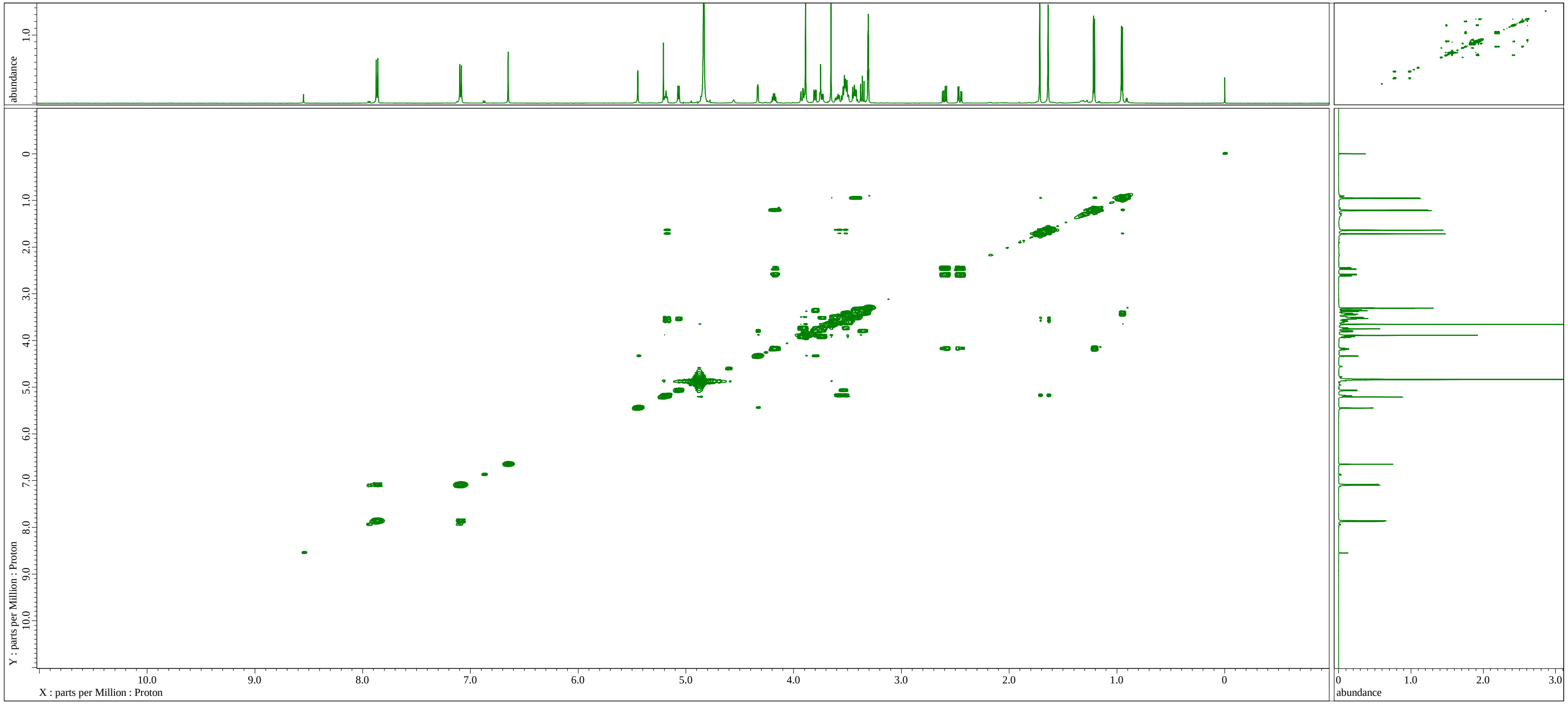

Figure S 3-6. The COSY spectrum of **2** in CD_3OD.

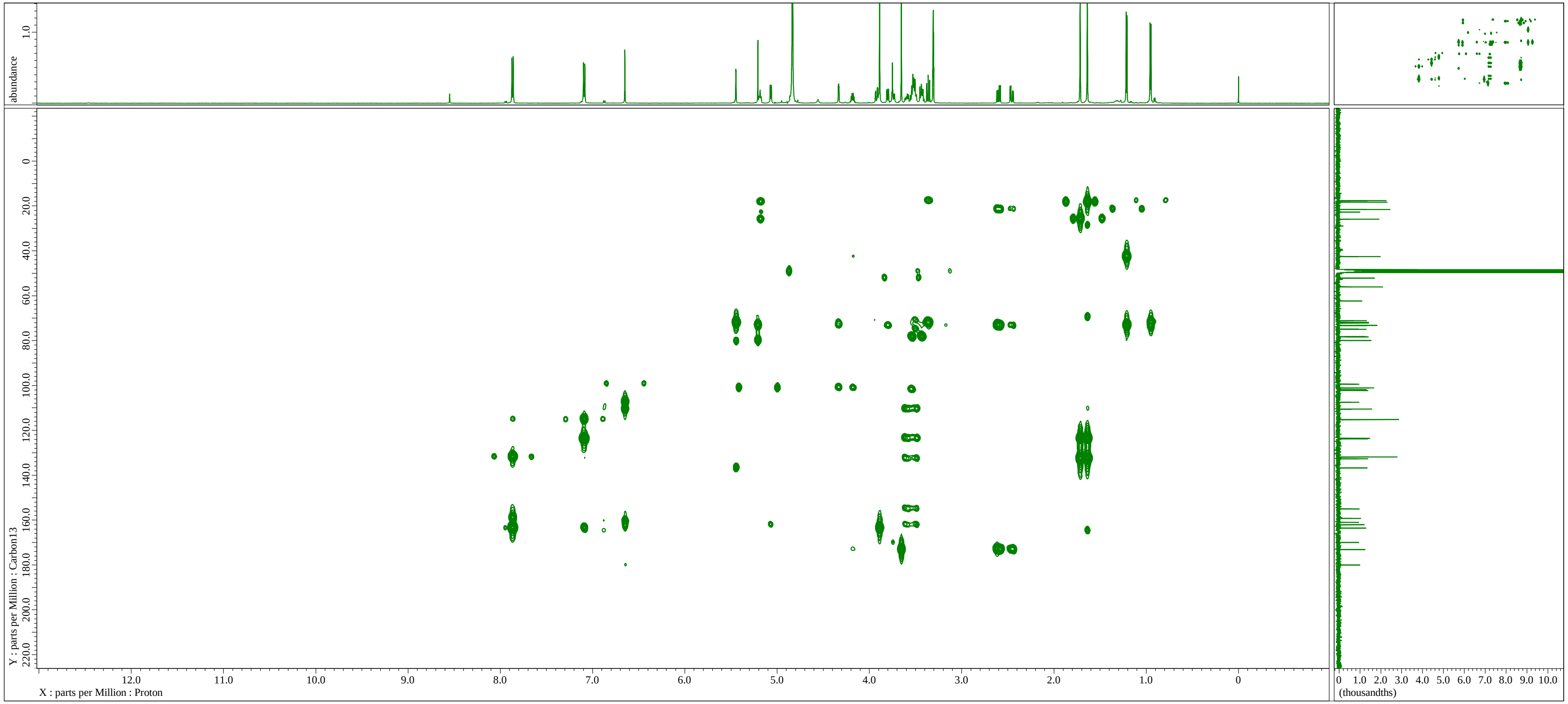

Figure S 3-7. The HMBC spectrum of **2** in CD_3OD.

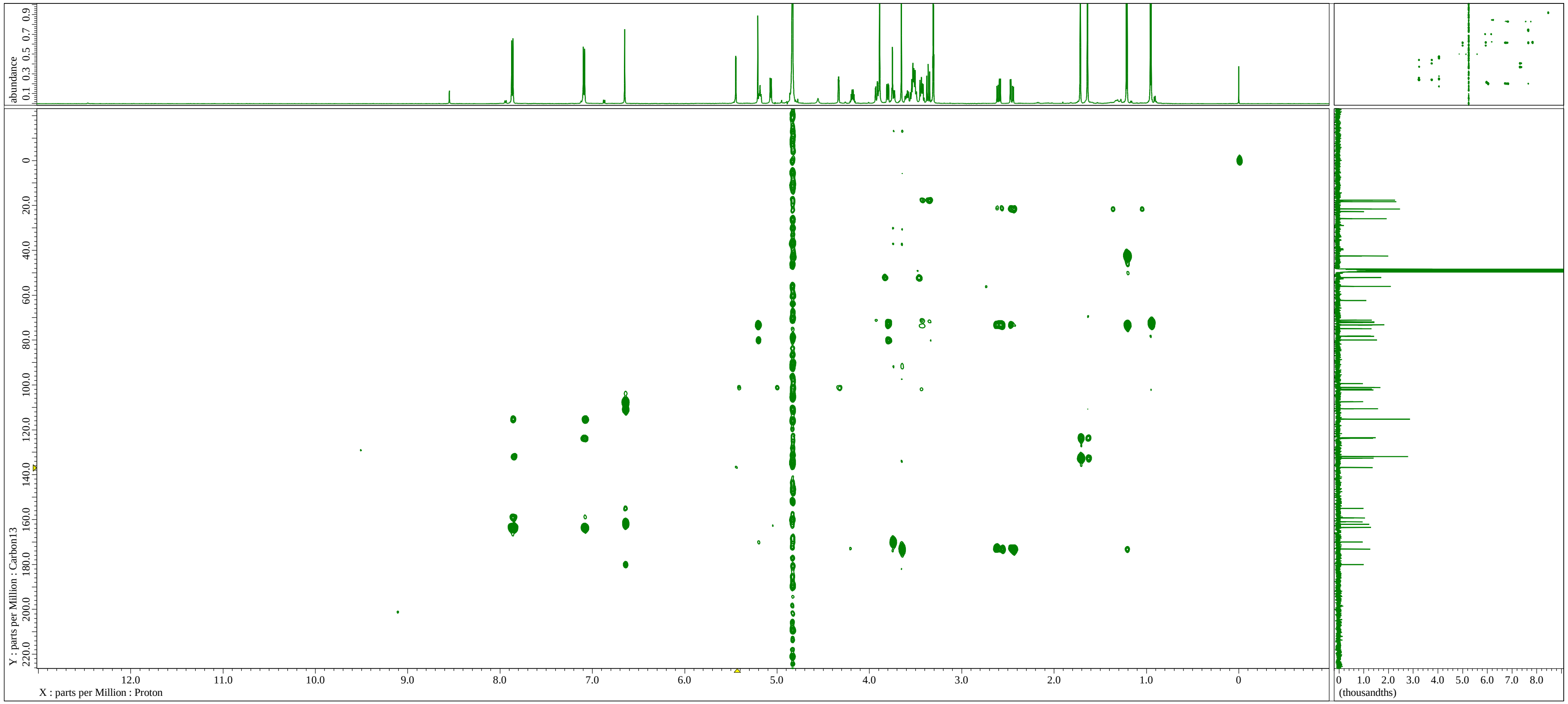

Figure S 3-8. The HMBC (2 Hz) spectrum of **2** in CD_3OD.

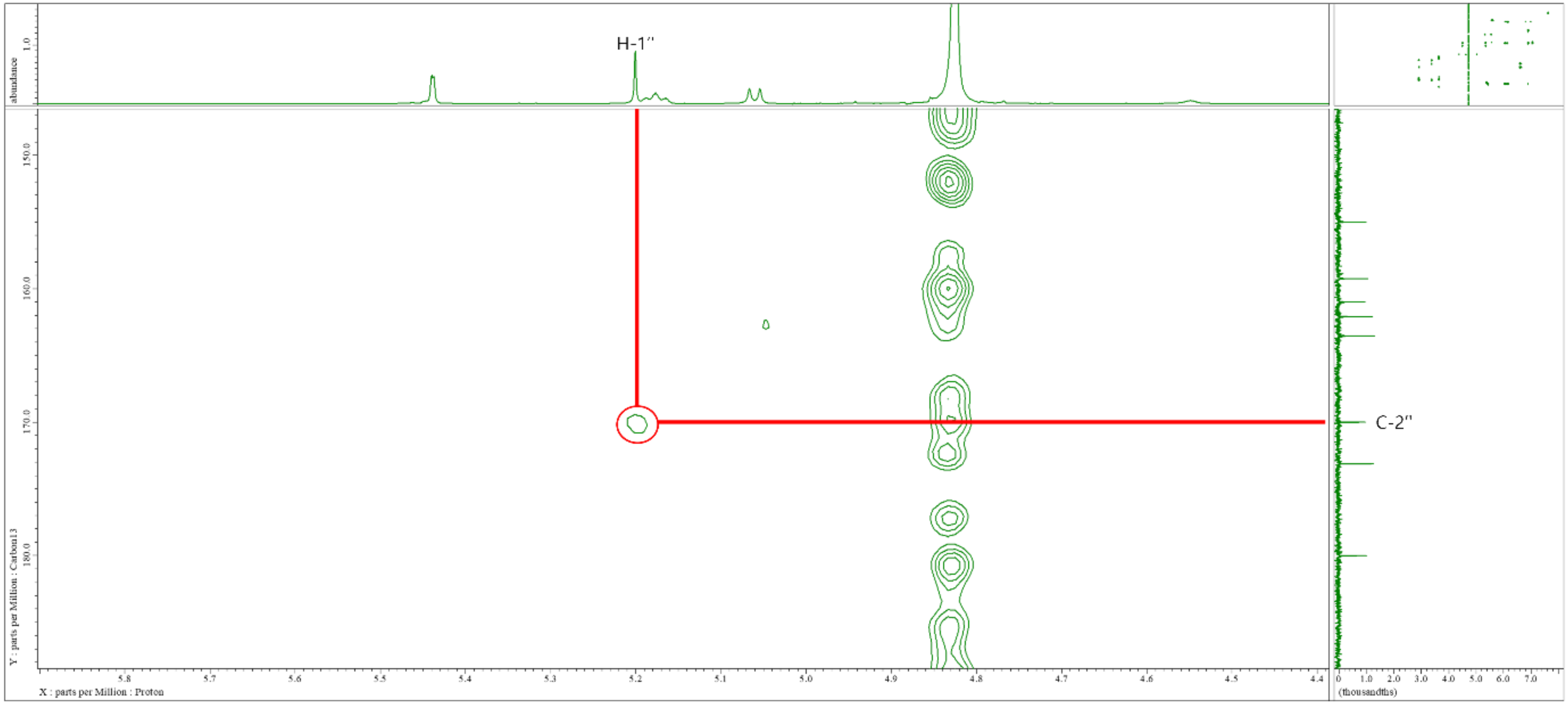

Figure S 3-8. The Expanded HMBC (2 Hz) spectrum of **2** in CD_3OD.

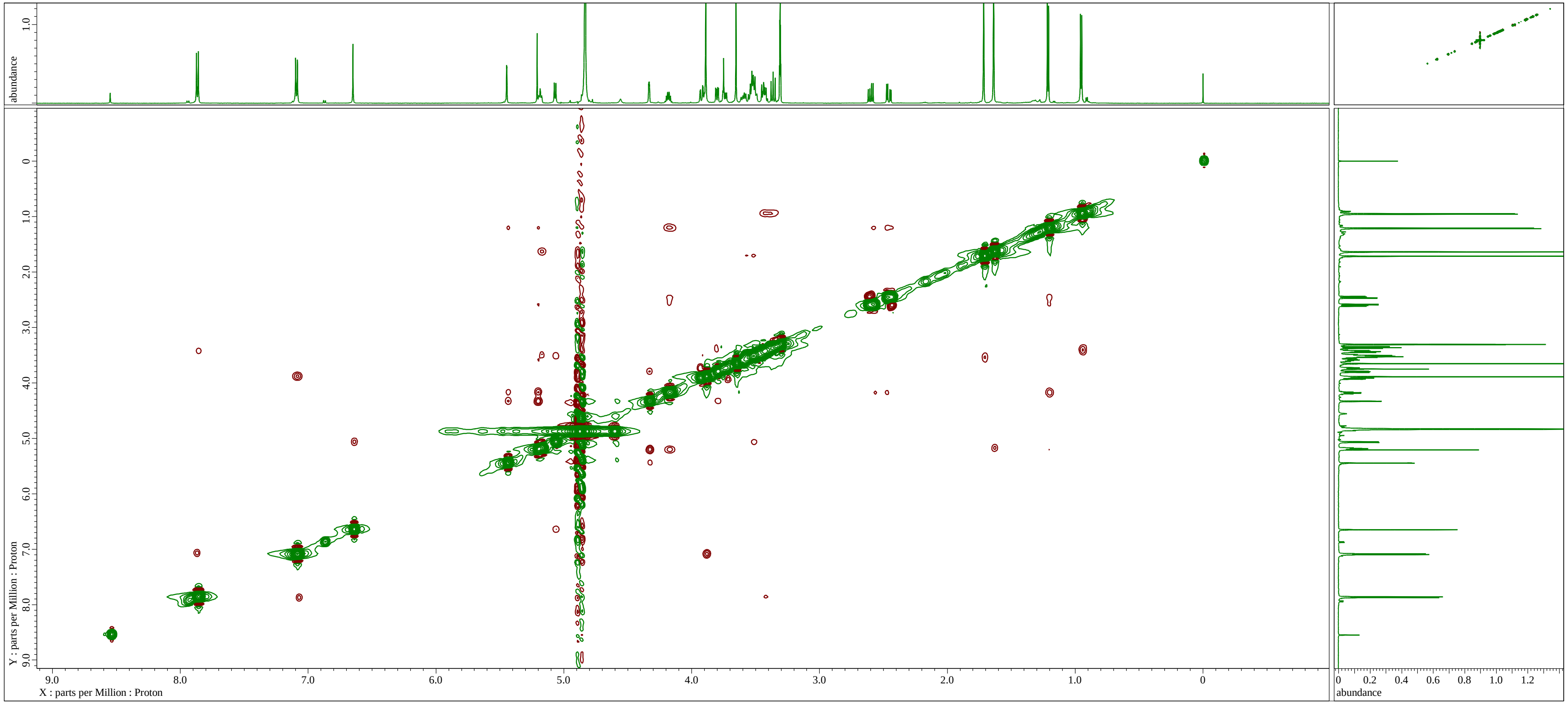

Figure S 3-9. The NOESY spectrum of **2** in CD_3OD.

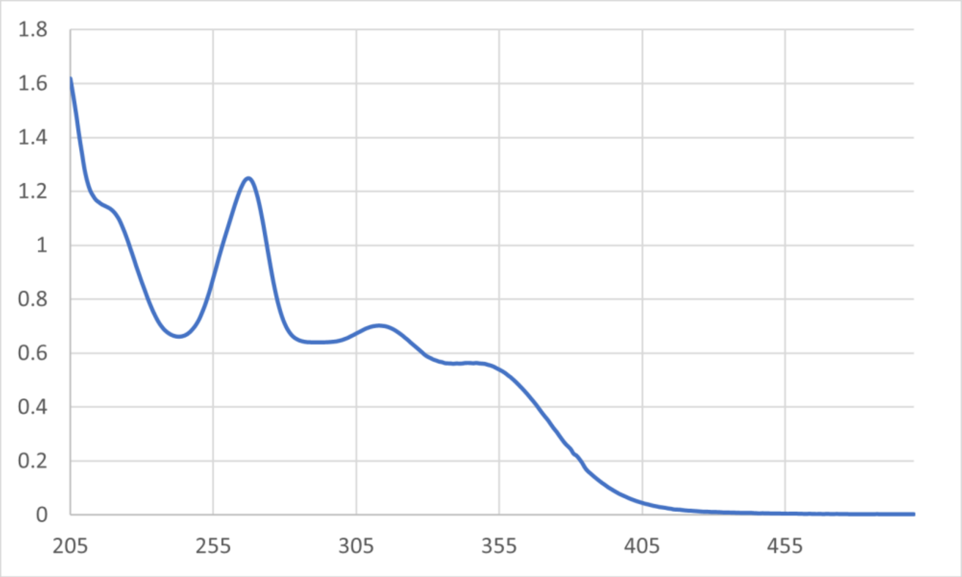

Figure S 3-10. The UV spectrum of **2** in CD_3OD.

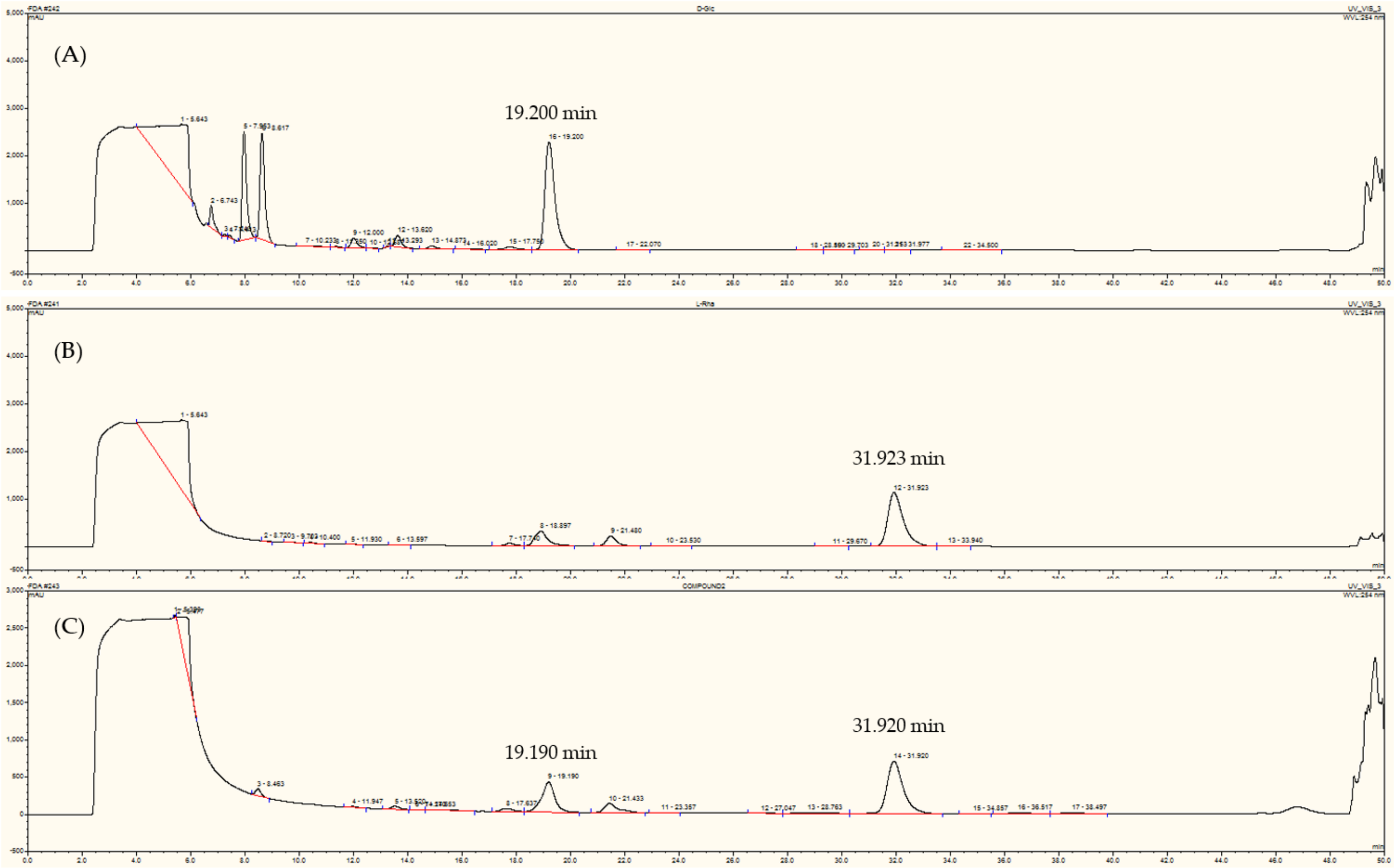

(A) D-glucose derivative (B) L-rhamnose derivative (C) derivatives of acid hydrolysate

Figure S 3-11. Acid hydrolysis of **2.**

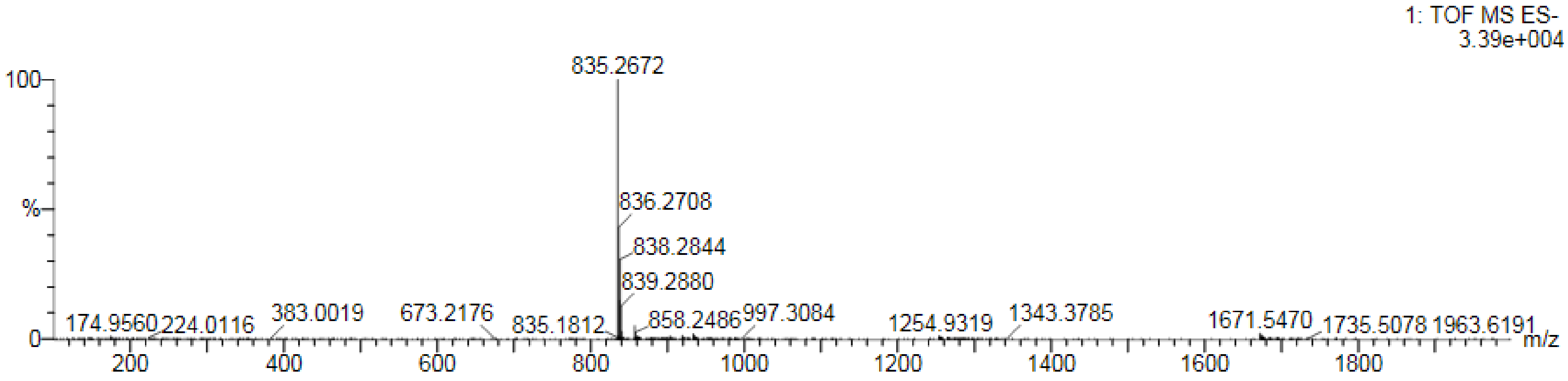

Minimum:				-1.5				
Maximum:		5.0	10.0	50.0				

Mass	Calc. Mass	mDa	PPM	DBE	i-FIT	Norm	Conf(%)	Formula
835.2672	835.2661	1.1	1.3	16.5	193.6	0.013	98.70	C39 H47 O20
	835.2696	-2.4	-2.9	38.5	200.0	6.412	0.16	C57 H39 O7
	835.2637	3.5	4.2	47.5	201.7	8.143	0.03	C64 H35 O2
	835.2719	-4.7	-5.6	7.5	199.0	5.447	0.43	C32 H51 O25
	835.2602	7.0	8.4	25.5	198.7	5.194	0.56	C46 H43 O15
	835.2755	-8.3	-9.9	29.5	200.3	6.727	0.12	C50 H43 O12

Figure S 4-1. The ESIHRMS of **3.**

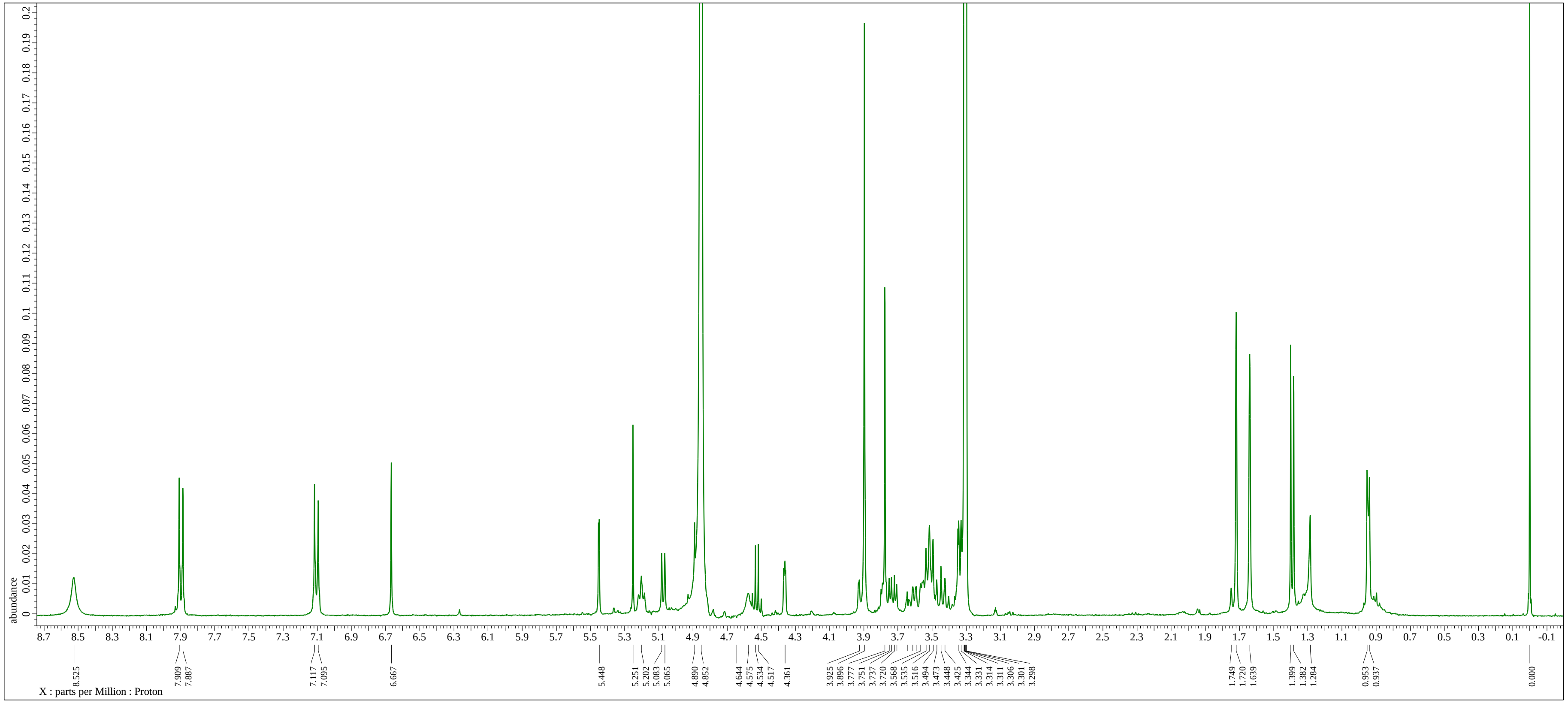

Figure S 4-2. The ^{1}H NMR (400 MHz) spectrum of **3** in CD_3OD.

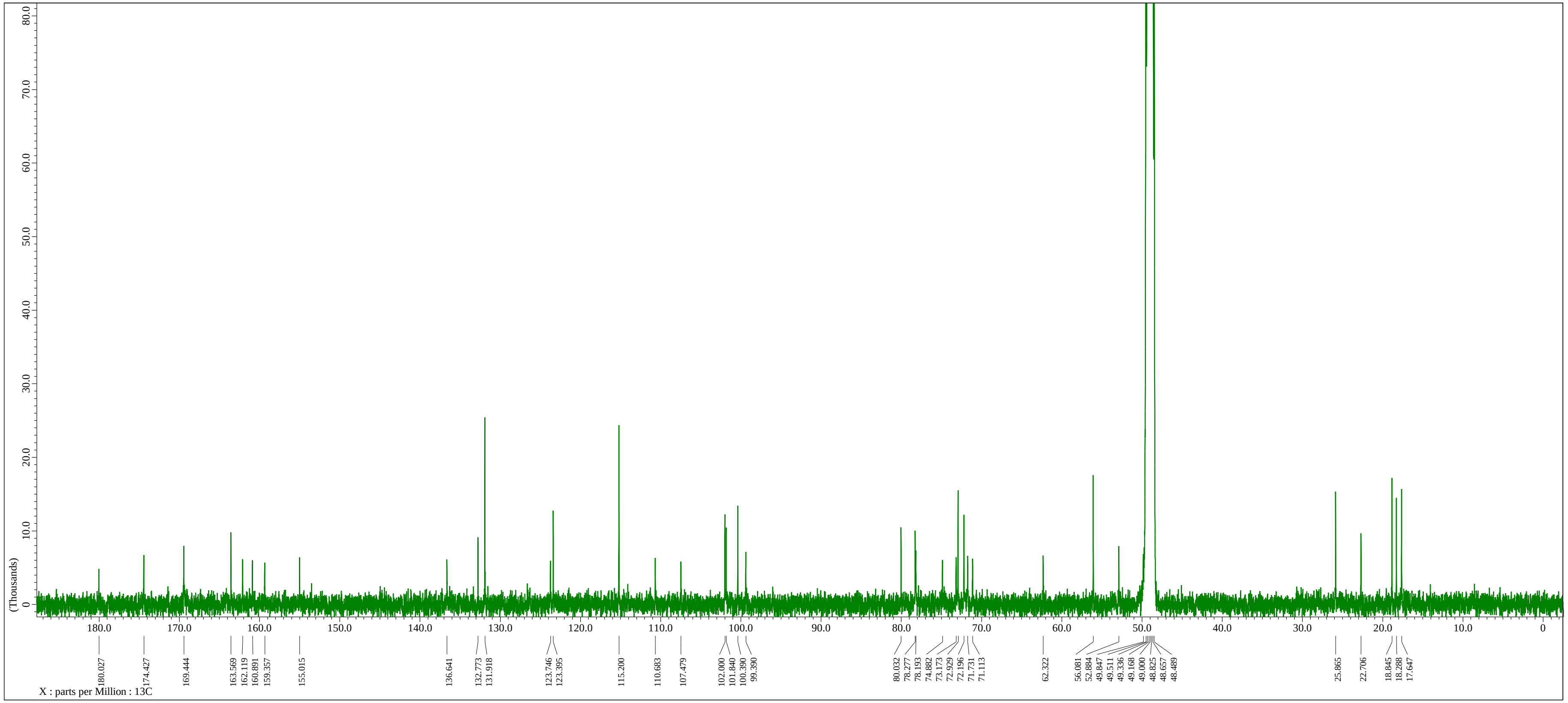

Figure S 4-3. The ^{13}C NMR (100 MHz) spectrum of **3** in CD_3OD.

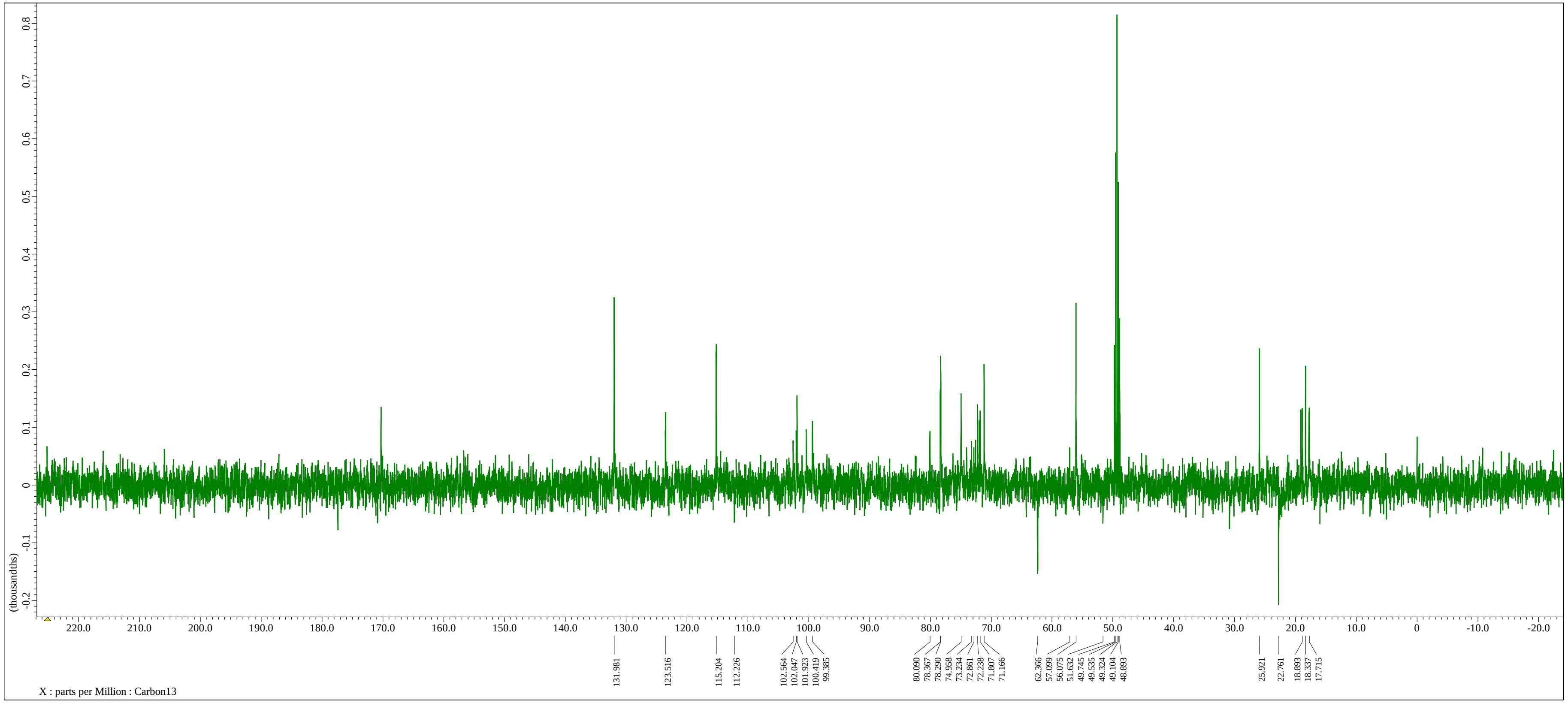

Figure S 4-4. The DEPT-135 spectrum of **3** in CD_3OD.

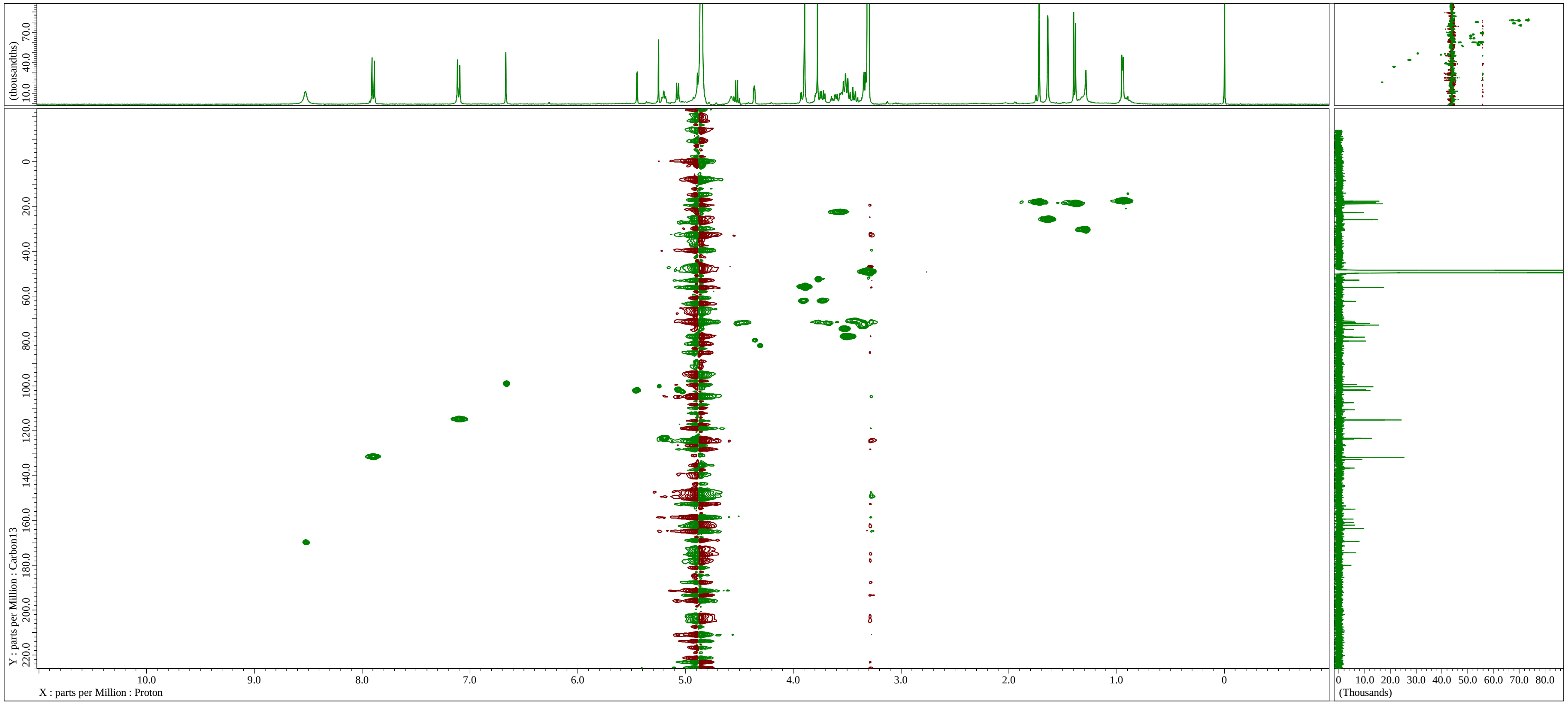

Figure S 4-5. The HSQC spectrum of **3** in CD_3OD.

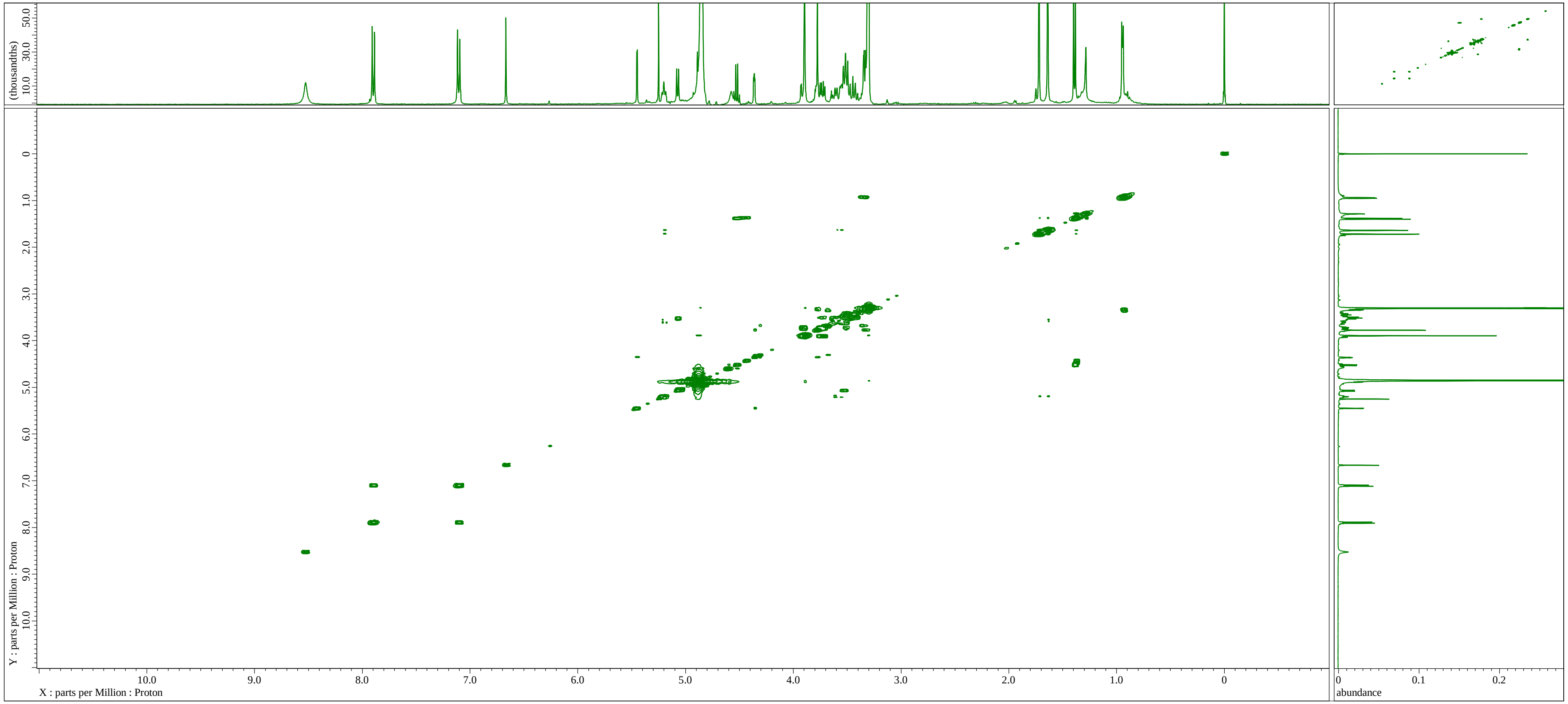

Figure S 4-6. The COSY spectrum of **3** in CD_3OD.

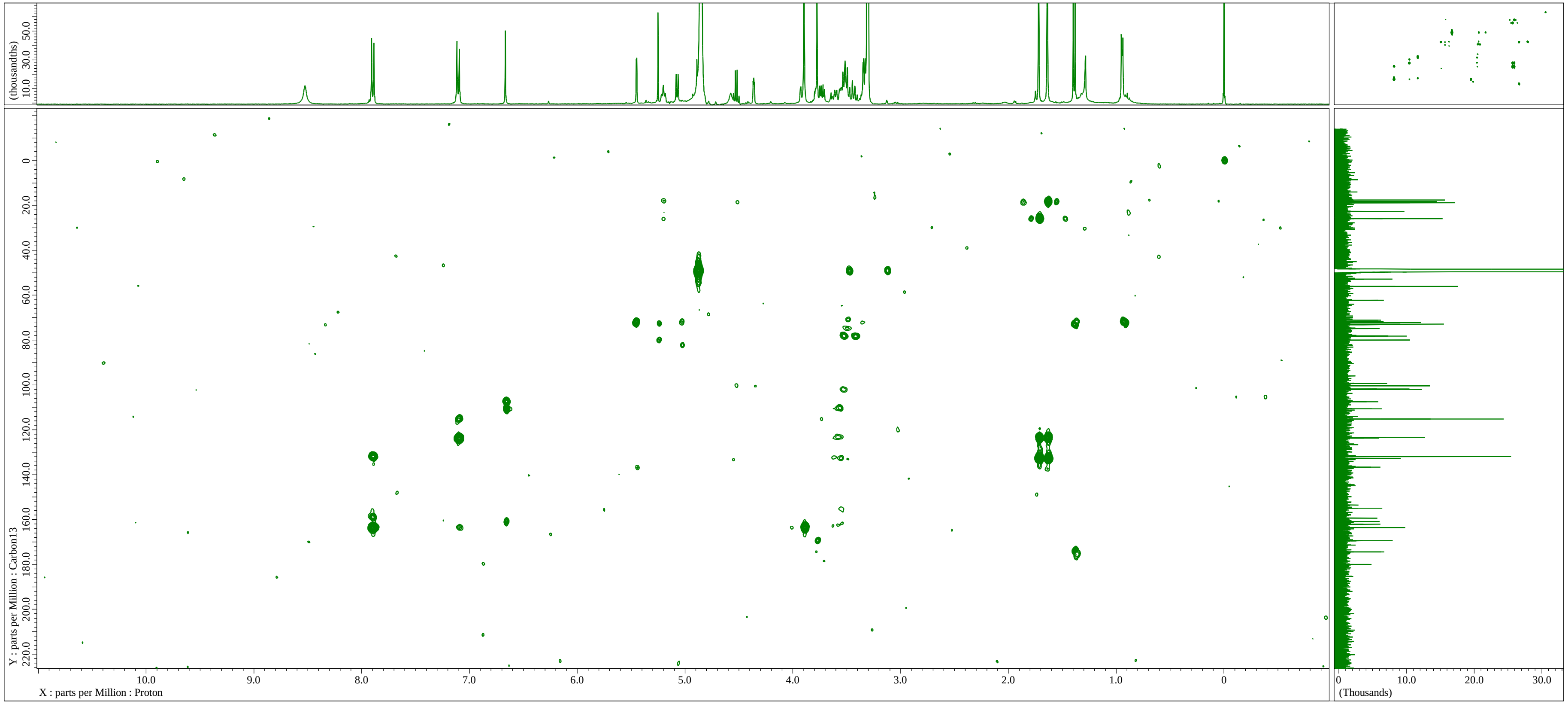

Figure S 4-7. The HMBC spectrum of **3** in CD_3OD.

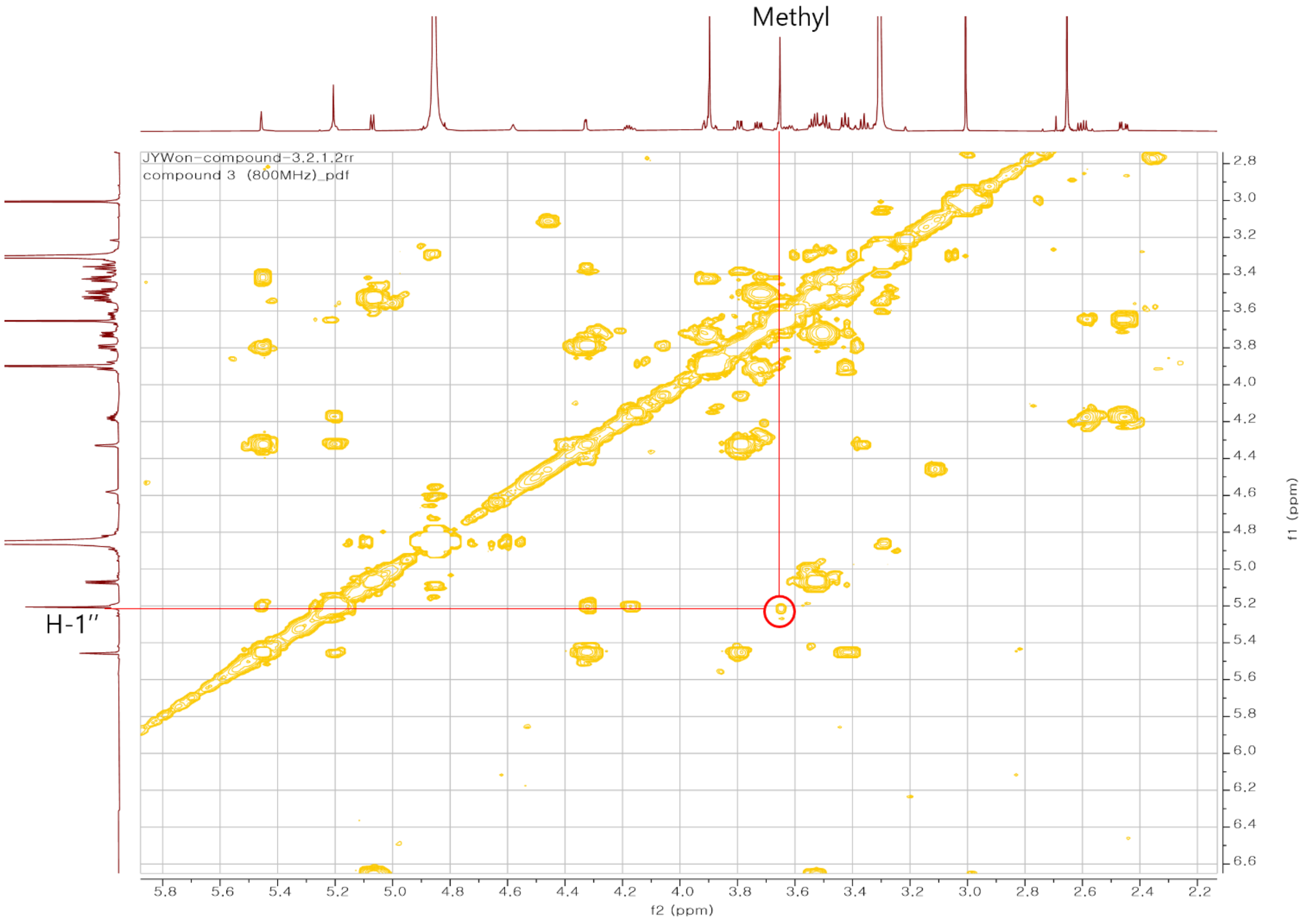

Figure S 4-8. The long-range COSY spectrum of **3** in CD_3OD.

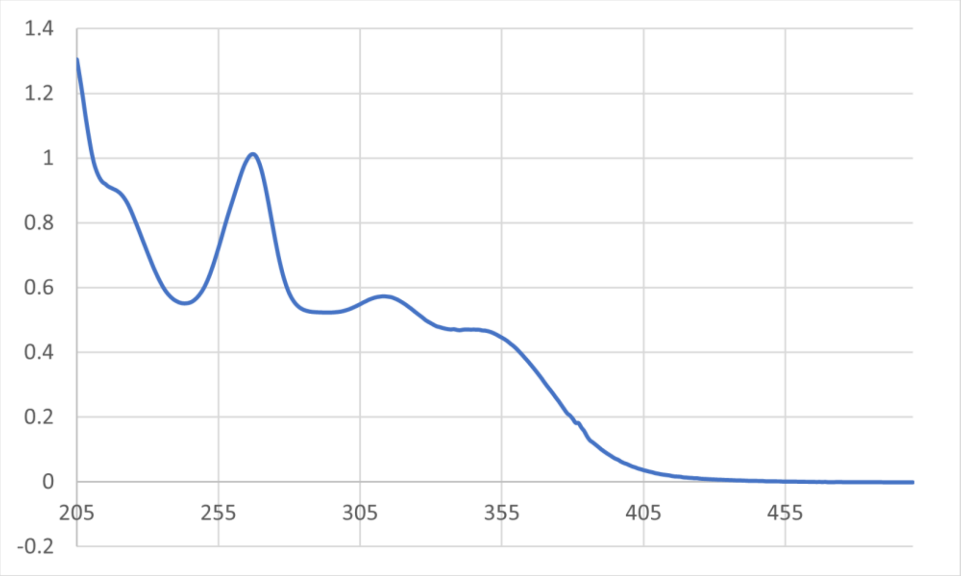

Figure S 4-9. The UV spectrum of **3** in CD_3OD.

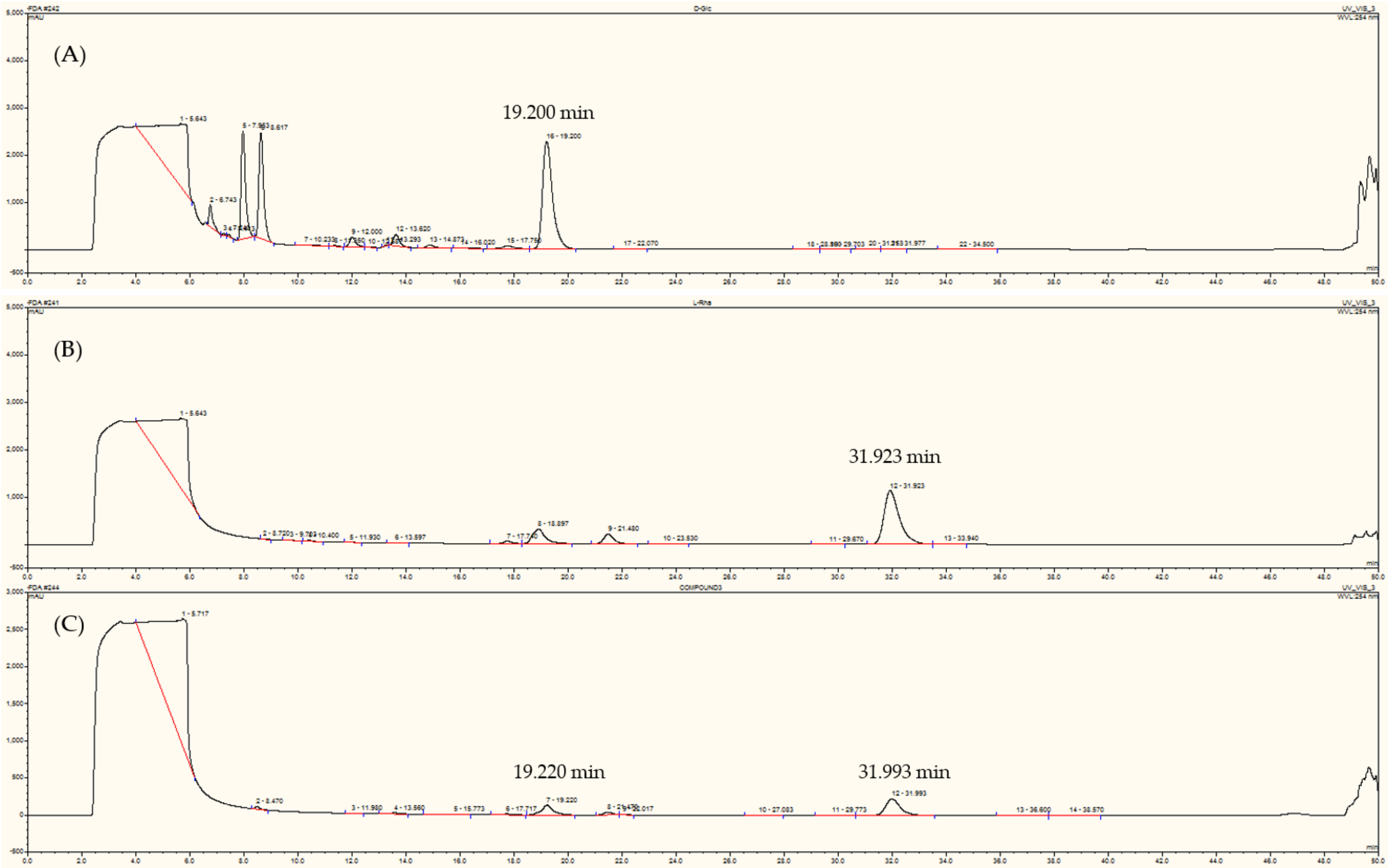

(A) D-glucose derivative (B) L-rhamnose derivative (C) derivatives of acid hydrolysate

Figure S 4-10. Acid hydrolysis of **3.**

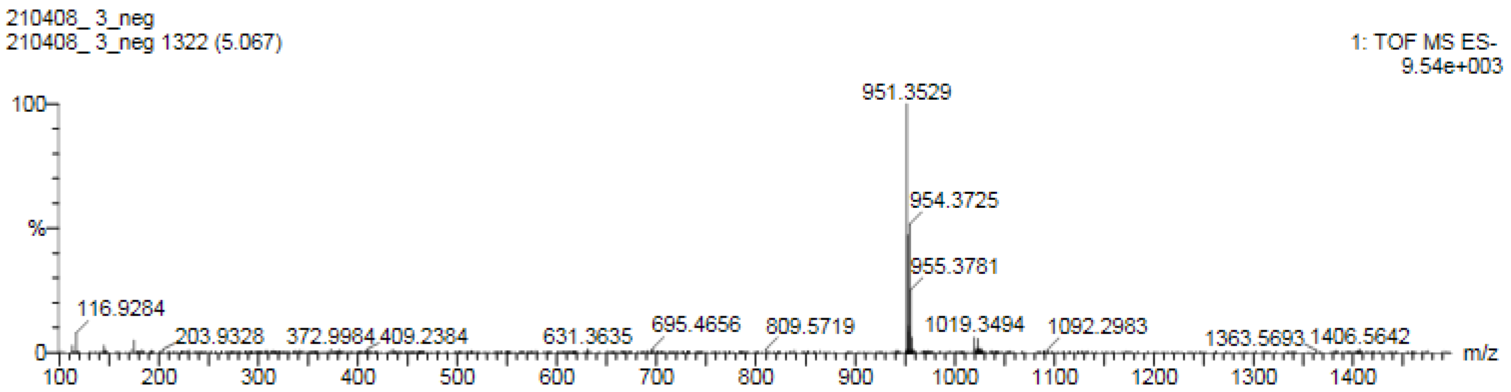

Minimum:				-1.5				
Maximum:		5.0	10.0	50.0				

Mass	Calc. Mass	mDa	PPM	DBE	i-FIT	Norm	Conf(%)	Formula
951.3529	951.3533	-0.4	-0.4	38.5	140.9	4.742	0.87	C63 H51 O9
	951.3557	-2.8	-2.9	7.5	136.8	0.645	52.46	C38 H63 O27
	951.3498	3.1	3.3	16.5	137.8	1.602	20.15	C45 H59 O22
	951.3474	5.5	5.8	47.5	141.7	5.512	0.40	C70 H47 O4
	951.3592	-6.3	-6.6	29.5	140.0	3.814	2.21	C56 H55 O14
	951.3615	-8.6	-9.0	-1.5	137.8	1.650	19.20	C31 H67 O32
	951.3439	9.0	9.5	25.5	139.2	3.058	4.70	C52 H55 O17

Figure S 5-1. The ESIHRMS of **4**.

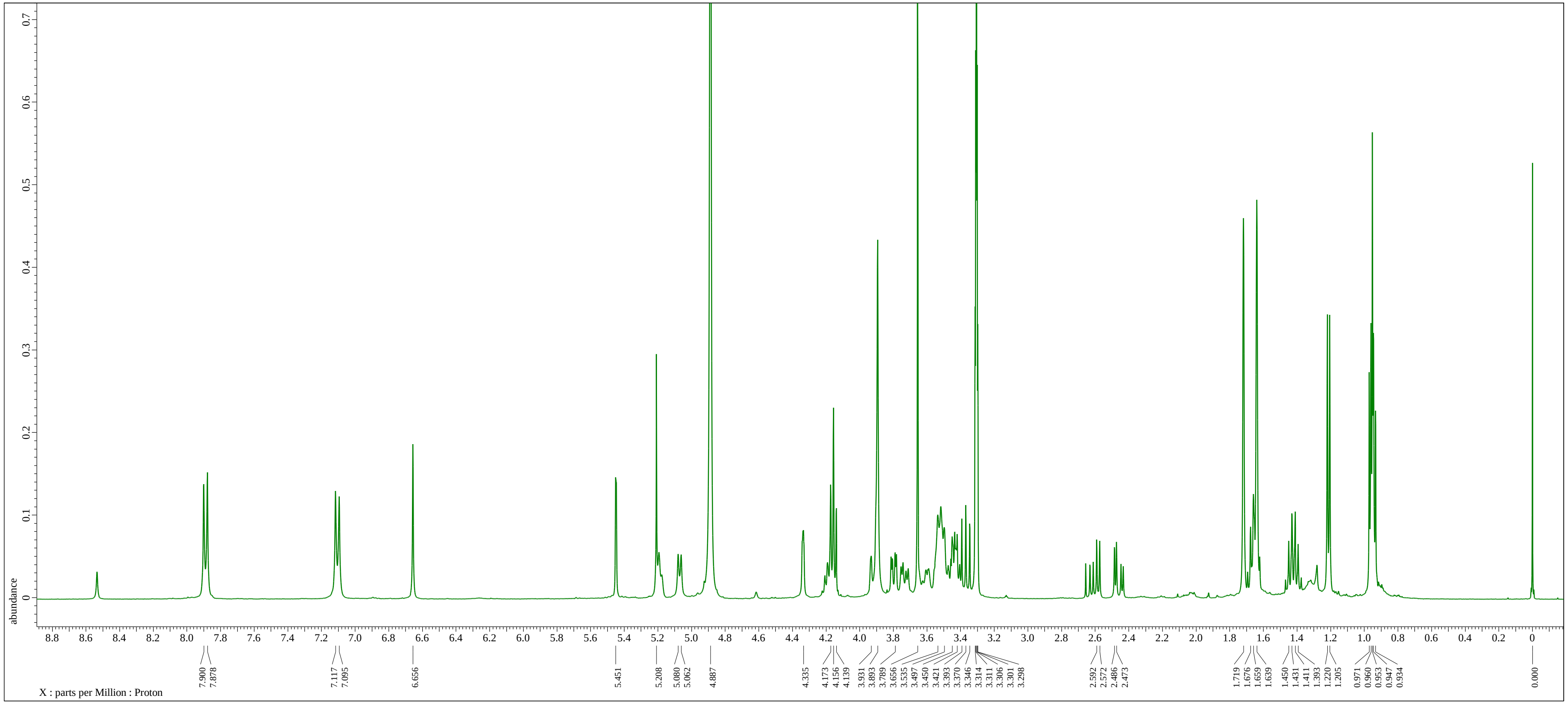

Figure S 5-2. The ¹H NMR (400 MHz) spectrum of **4** in CD_3OD.

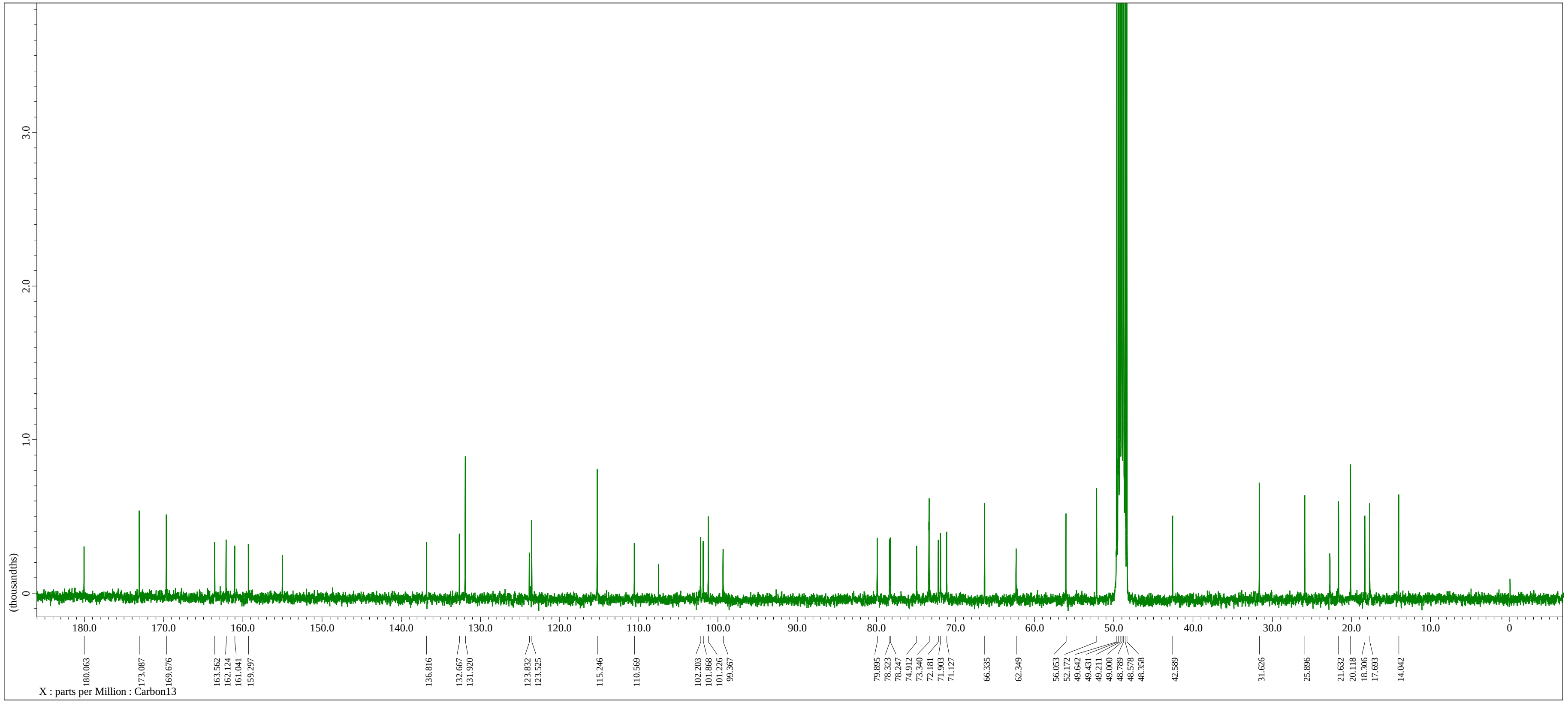

Figure S 5-3. The ^{13}C NMR (100 MHz) spectrum of **4** in CD_3OD.

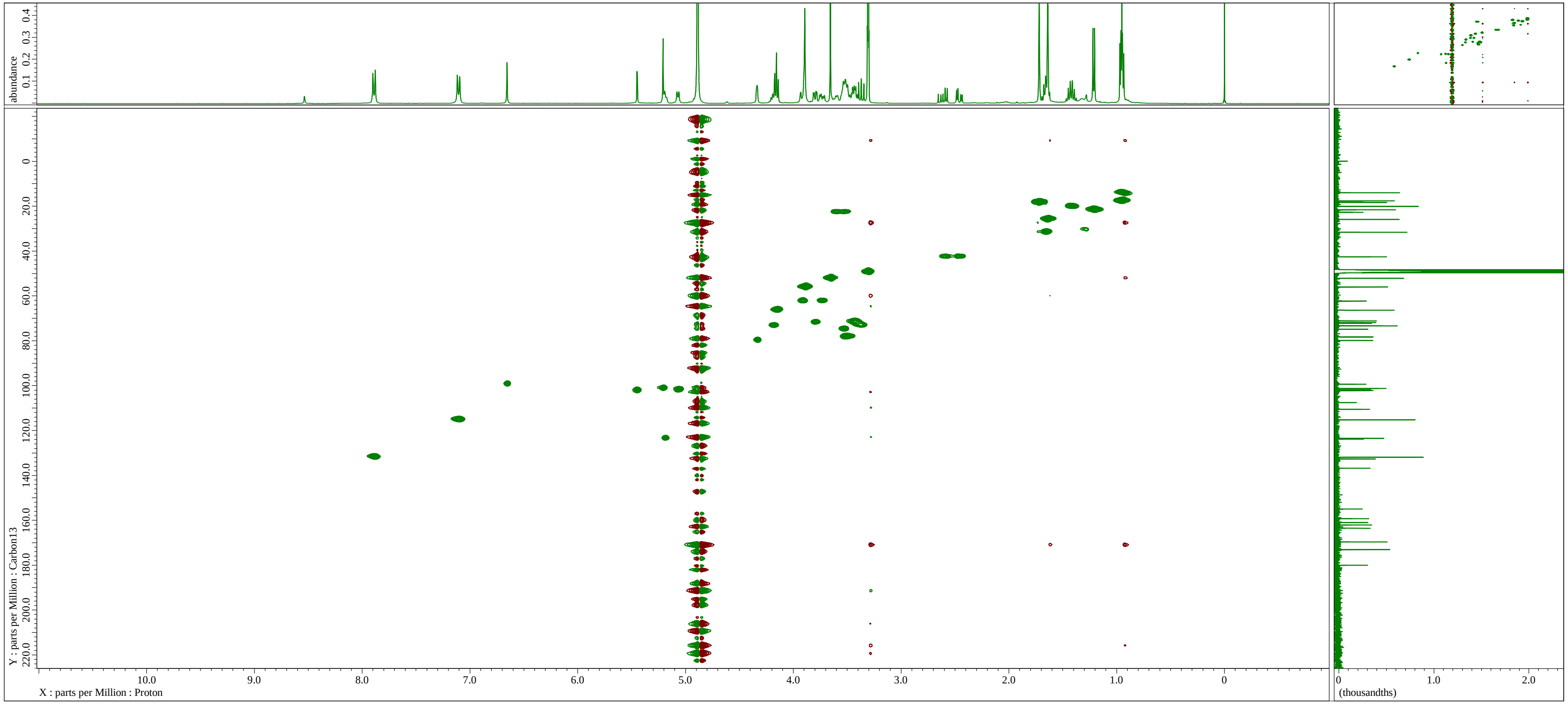

Figure S 5-4. The HSQC spectrum of **4** in CD_3OD.

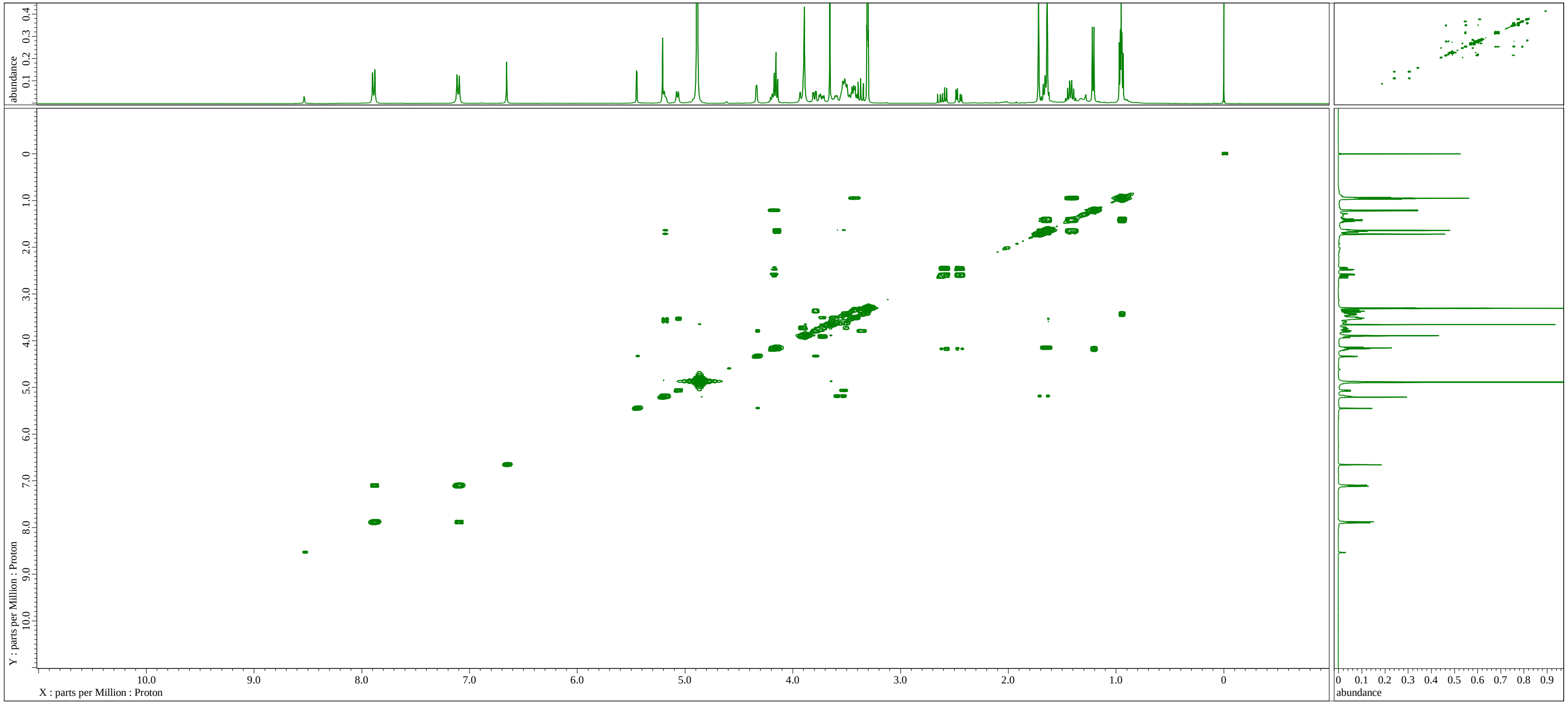

Figure S 5-5. The COSY spectrum of **4** in CD_3OD.

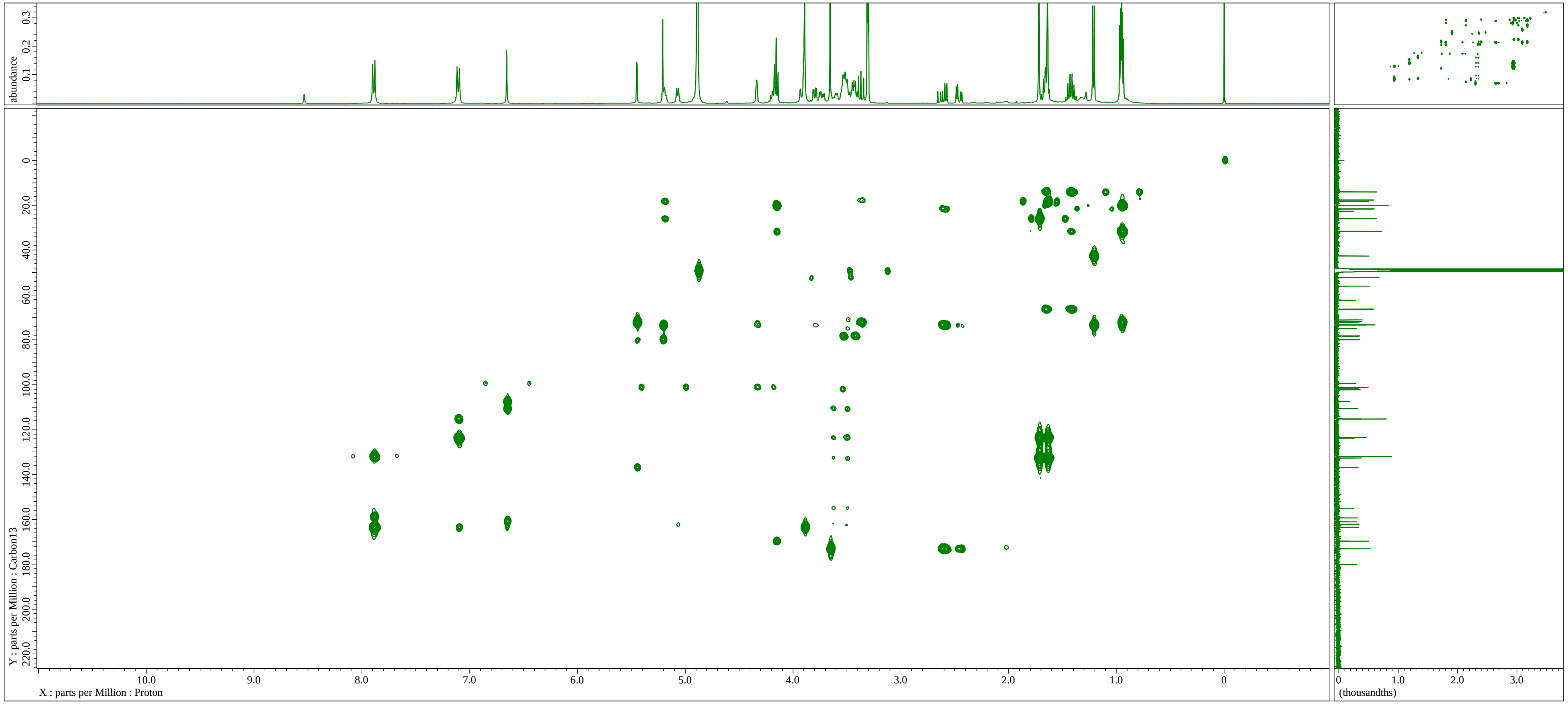

Figure S 5-6. The COSY spectrum of **4** in CD_3OD.

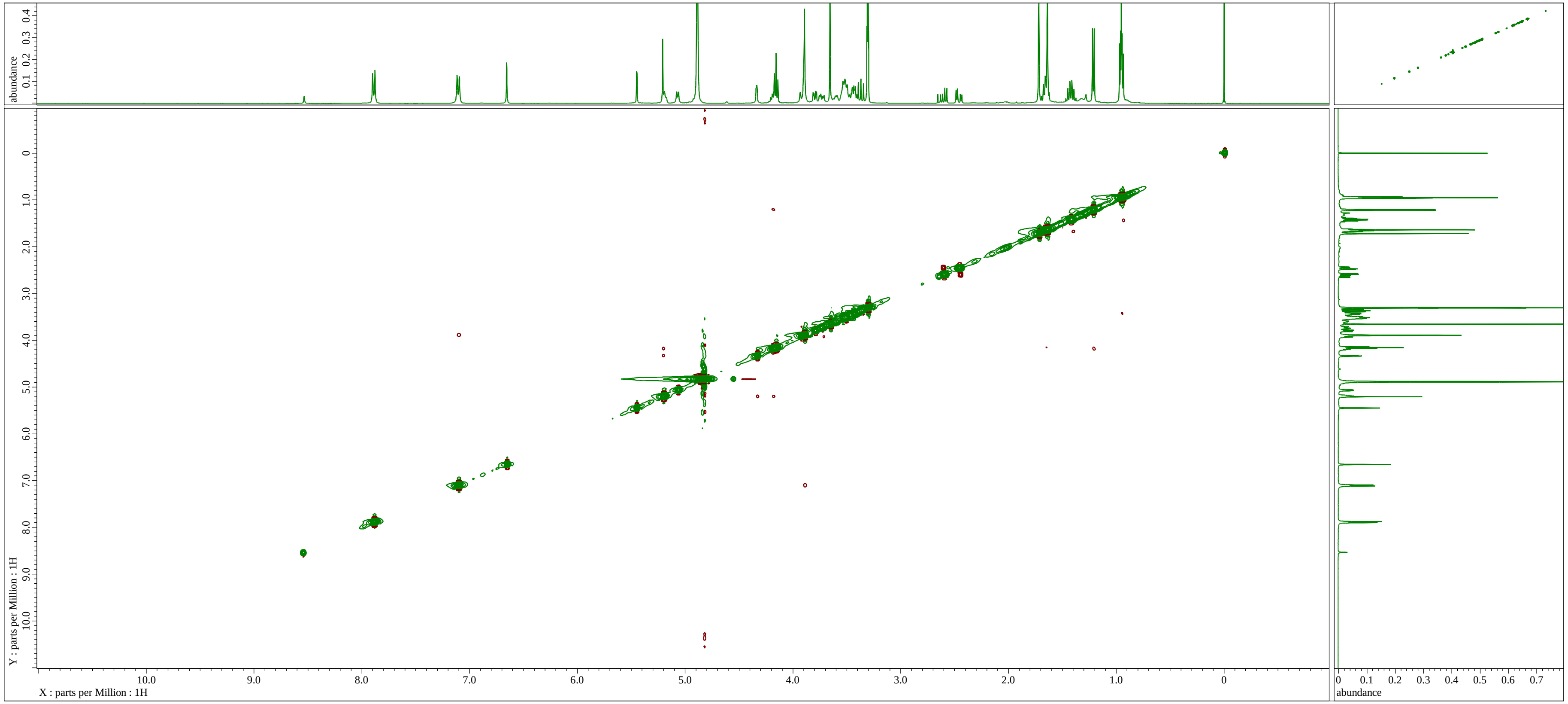

Figure S 5-7. The NOESY spectrum of **4** in CD_3OD.

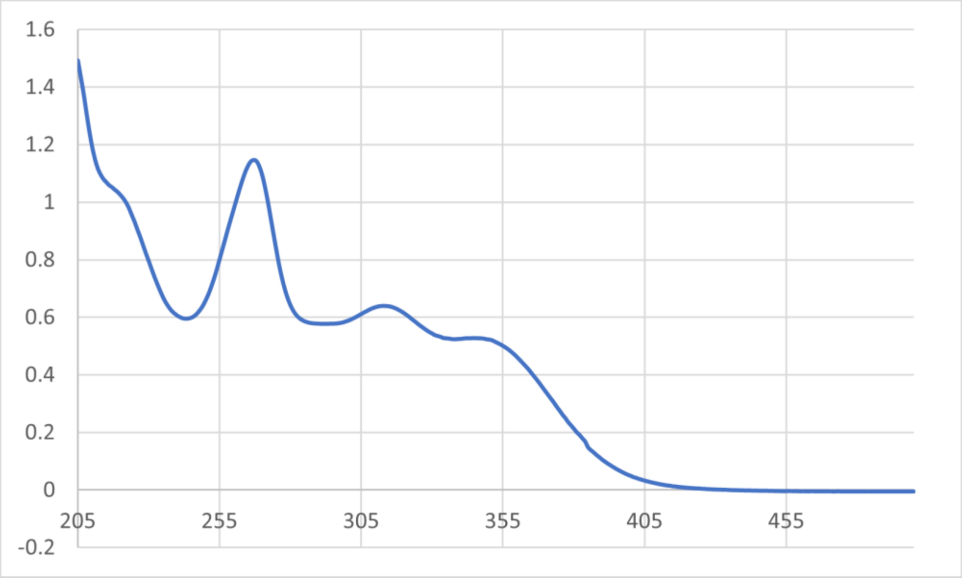

Figure S 5-8. The UV spectrum of **4**.

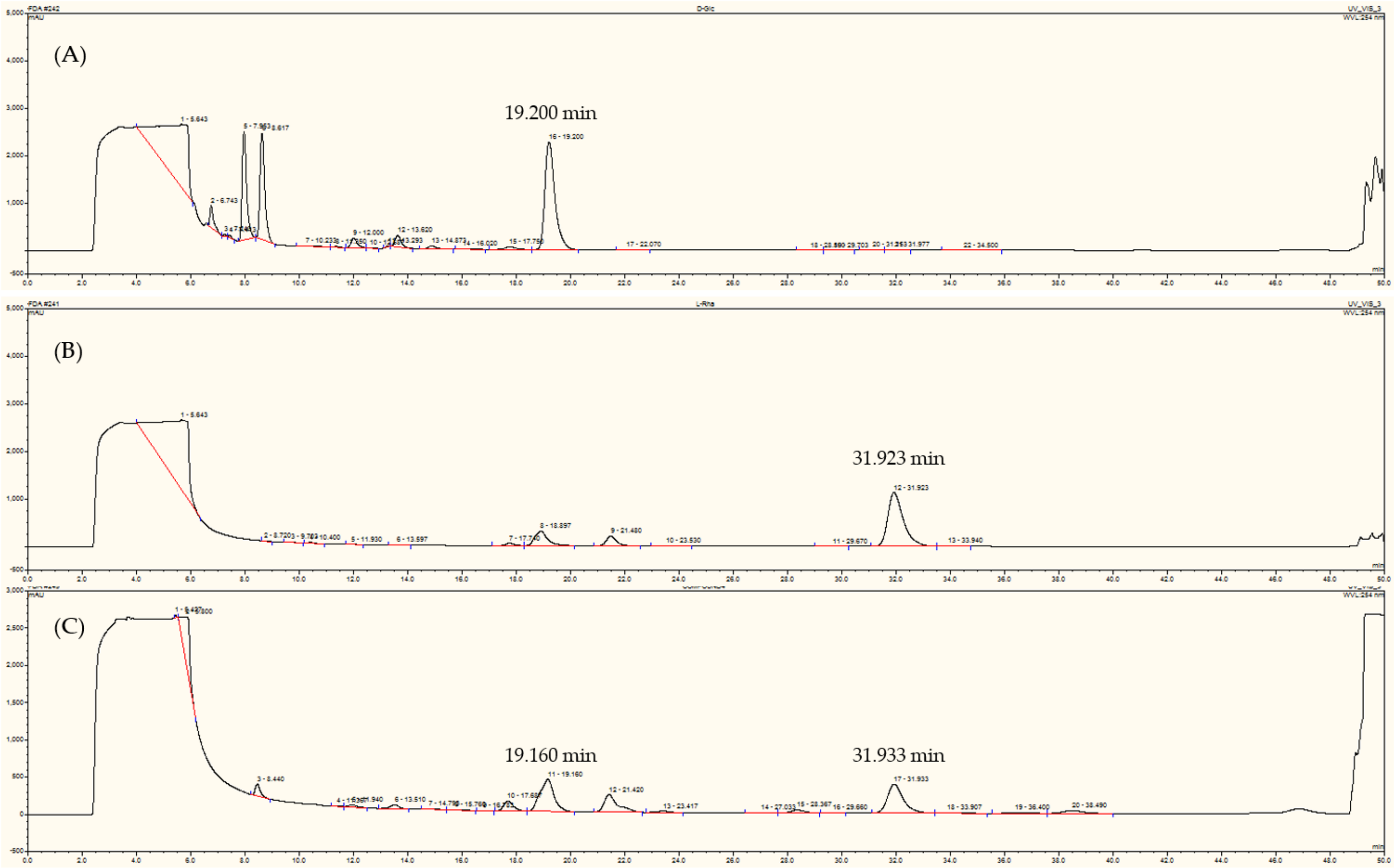

(A) D-glucose derivative (B) L-rhamnose derivative (C) derivatives of acid hydrolysate

Figure S 5-9. Acid hydrolysis of **4.**

Table S 6 – 1. Assignment of ^{1}H and ^{13}C NMR spectra of **7** in CD_3OD

	7	
**Position**	**δ_H (*J* in Hz)**	**δ_C**
2		159.2
3		136.1
4		179.9
5		160.8
6	6.25, s	99.3
7		163.2
8		107.9
9		155.8
10		106.0
11	3.41, m<br>3.46, m	22.5
12	5.18, t (6.8)	123.8
13		132.5
14	1.66, s	25.9
15	1.72, s	18.2
1′		122.9
2′, 6′	7.78, d (8.8)	132.0
3′, 5′	6.94, d (8.8)	116.5
4′		161.6
Rhamnose		
1	5.37, brs	103.6
2	4.23, brs	72.0
3	3.72, m	72.08
4	3.33, m	72.15
5	3.35, m	73.2
6	0.92, d (5.6)	17.7

^{1}H and ^{13}C-NMR spectra were obtained with a Jeol 400 (JEOL, Tokyo, Japan)

The ^{13}C-NMR spectrum showed 26 carbon atoms. Its molecular formula was determined to be $C_{26}H_{28}O_{10}$ by negative mode high resolution ESI-QTOF-MS based on the ion peak at *m*/*z* 499.1598 $[M - H]^-$ (calcd. for $C_{26}H_{27}O_{10}$, 499.1598). A typical flavonol 3-glycoside skeleton was suggested by C-2 (δ_C 159.2), C-3 (δ_C 136.8) and α, β-unsaturated carbonyl group (C-4, δ_C 180.0). The ^{1}H NMR spectrum showed the presence of a prenyl group at δ_H 1.66 (3H, s, H-14) ,1.72 (3H, s, H-15), 3.41 (1H, m, H-11a), 3.46 (1H, m, H-11b) and 5.18 (1H, t, *J* = 6.8 Hz, H-12), a anomeric proton at δ_H 5.37 (1H, brs, Rha H-1), a *penta*-substituted benzen ring at δ_H 6.25 (1H, s, H-6) and a *para*-substituted benzen ring at δ_H 6.94 (2H, d, *J* = 8.8 Hz, H-3′, 5′) and 7.78 (2H, d, *J* = 8.8 Hz, H-2′, 6′). The location of prenyl group at the C-8 (δ_C 107.9) was supported by the downfiled shift of the carbon atom at C-8. The sugar moeity of **7** was identified as rhamonose by the anomeric proton at δ_H 5.37 (1H, brs, Rha H-1) and the ^{13}C NMR spectra at δ_C 103.6, 72.0, 72.08, 72.15, 73.2 and 17.7 (Rha C-1-6).

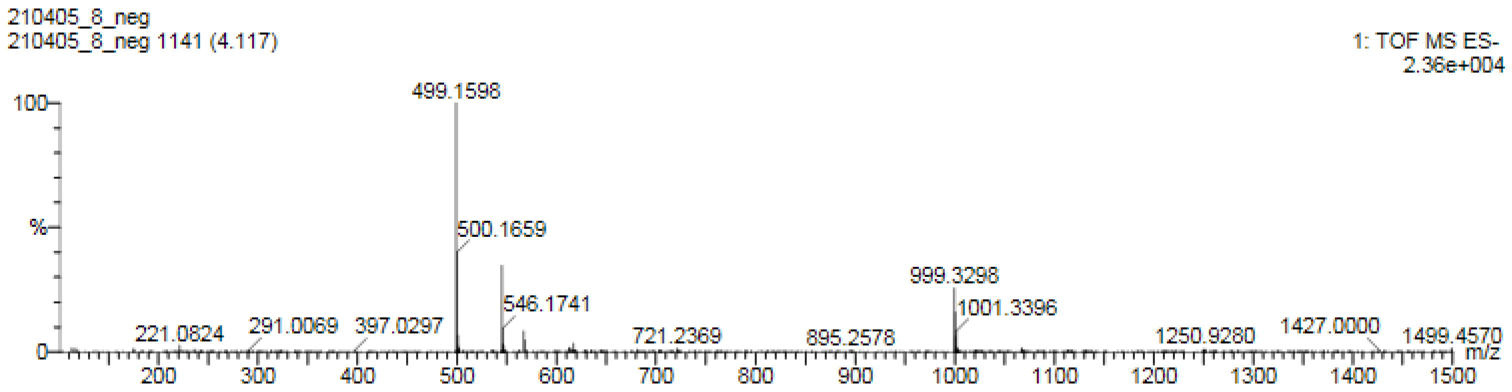

Minimum:				-1.5				
Maximum:		5.0	10.0	50.0				
Mass	Calc. Mass	mDa	PPM	DBE	i-FIT	Norm	Conf(%)	Formula
499.1598	499.1604	-0.6	-1.2	13.5	179.3	n/a	n/a	C26 H27 O10

Figure S 6 – 1. The ESIHRMS of **7**.

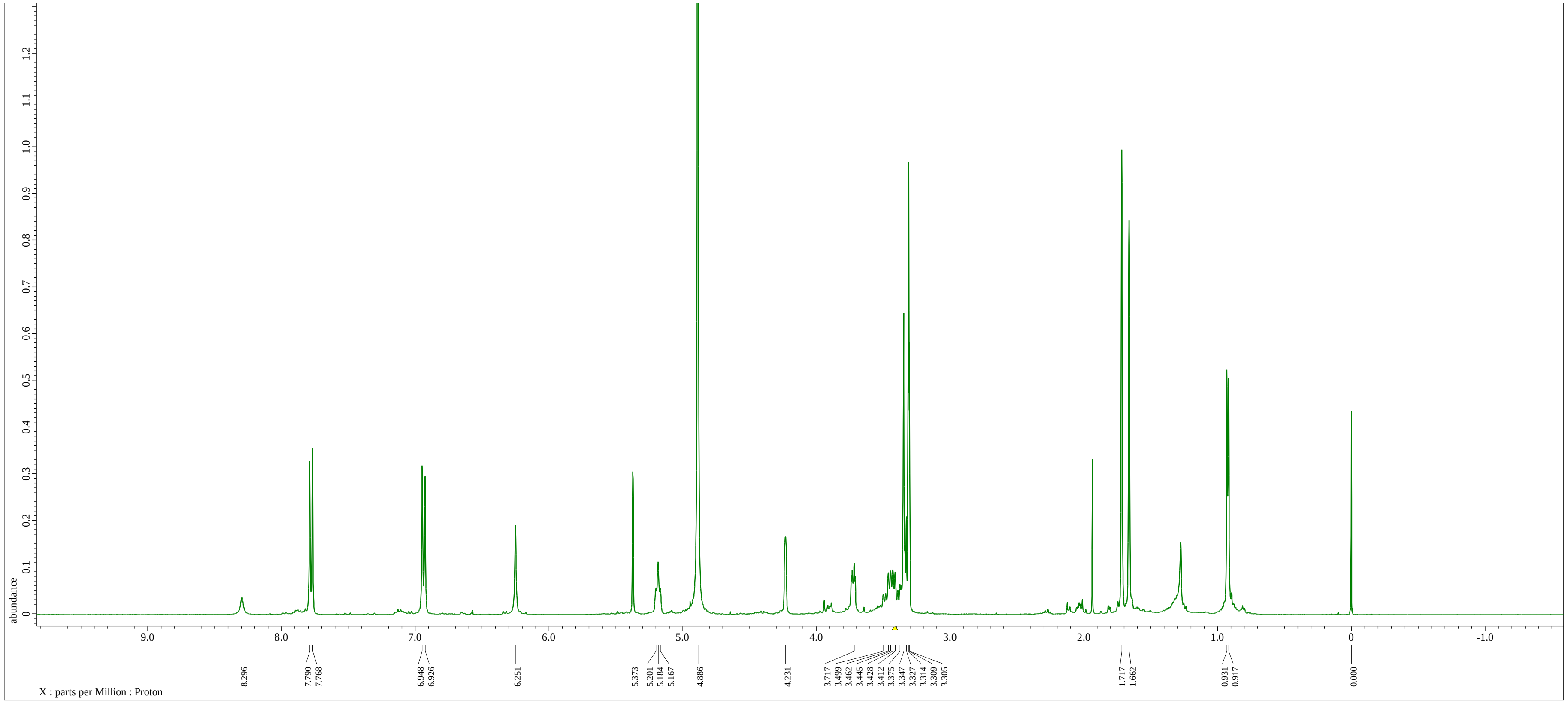

Figure S 6 – 2. The ^{1}H NMR (400 MHz) spectrum of **7** in CD_3OD.

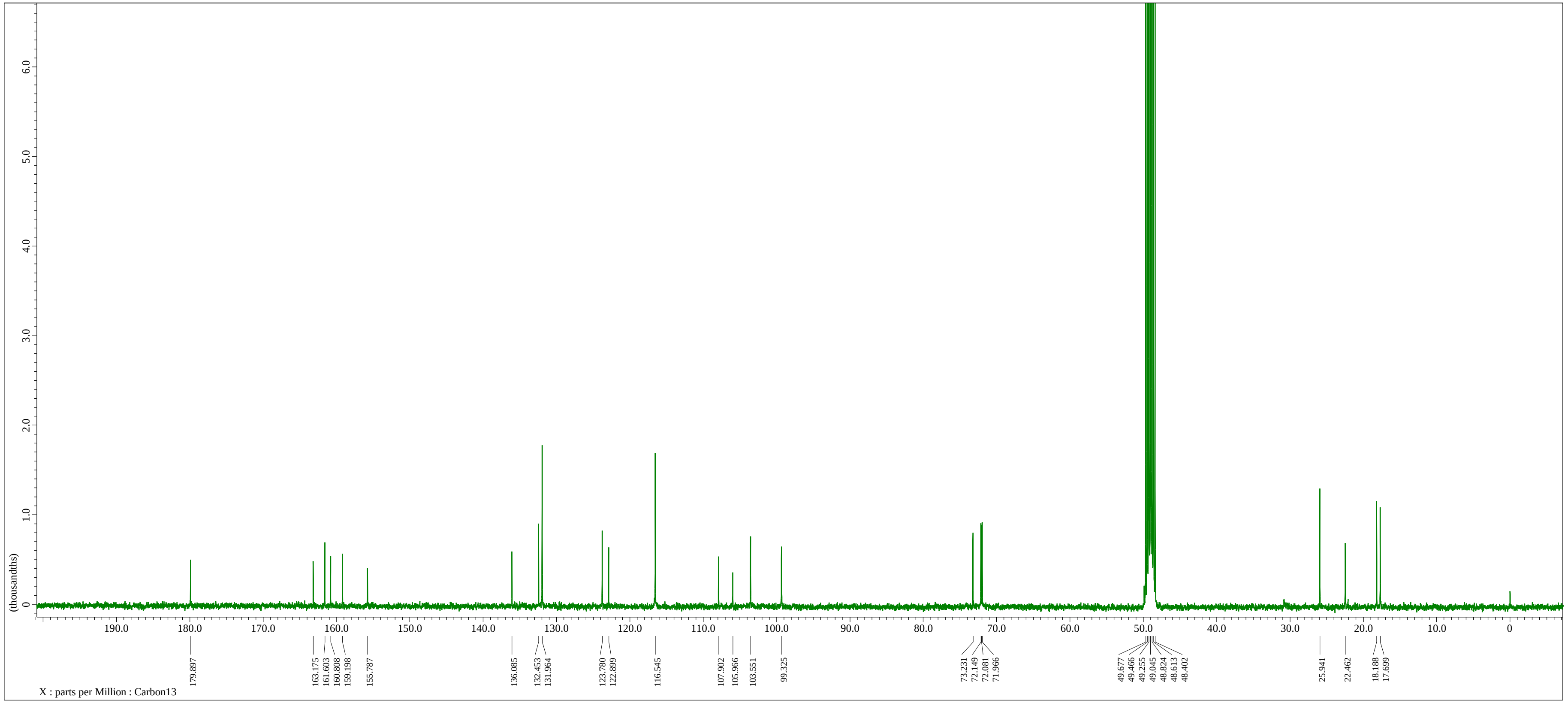

Figure S 6 – 3. The ^{13}C NMR (100 MHz) spectrum of **7** in CD_3OD.
